# Organometallic Routes into the Nanorealms of Binary Fe-Si Phases

**DOI:** 10.3390/ma3021049

**Published:** 2010-02-09

**Authors:** Manoj K. Kolel-Veetil, Teddy M. Keller

**Affiliations:** Advanced Materials Section, Chemistry Division, Naval Research Laboratory, Washington, DC 20375, USA; E-Mail: teddy.keller@nrl.navy.mil (T.M.K.)

**Keywords:** iron, silicide, organometallic, Fe_5_Si_3_, Fe_3_Si, FeSi, FeSi_2_, ferromagnet, Kondo, GMR, magnetoresistance, SLS, VLS

## Abstract

The Fe-Si binary system provides several iron silicides that have varied and exceptional material properties with applications in the electronic industry. The well known Fe-Si binary silicides are Fe_3_Si, Fe_5_Si_3_, FeSi, *α*-FeSi_2_ and *β*-FeSi_2_. While the iron-rich silicides Fe_3_Si and Fe_5_Si_3_ are known to be room temperature ferromagnets, the stoichiometric FeSi is the only known transition metal Kondo insulator. Furthermore, Fe_5_Si_3_ has also been demonstrated to exhibit giant magnetoresistance (GMR). The silicon-rich *β*-FeSi_2_ is a direct band gap material usable in light emitting diode (LED) applications. Typically, these silicides are synthesized by traditional solid-state reactions or by ion beam-induced mixing (IBM) of alternating metal and silicon layers. Alternatively, the utilization of organometallic compounds with reactive transition metal (Fe)-carbon bonds has opened various routes for the preparation of these silicides and the silicon-stabilized bcc- and fcc-Fe phases contained in the Fe-Si binary phase diagram. The unique interfacial interactions of carbon with the Fe and Si components have resulted in the preferential formation of nanoscale versions of these materials. This review will discuss such reactions.

## 1. Introduction

**Figure 1:**
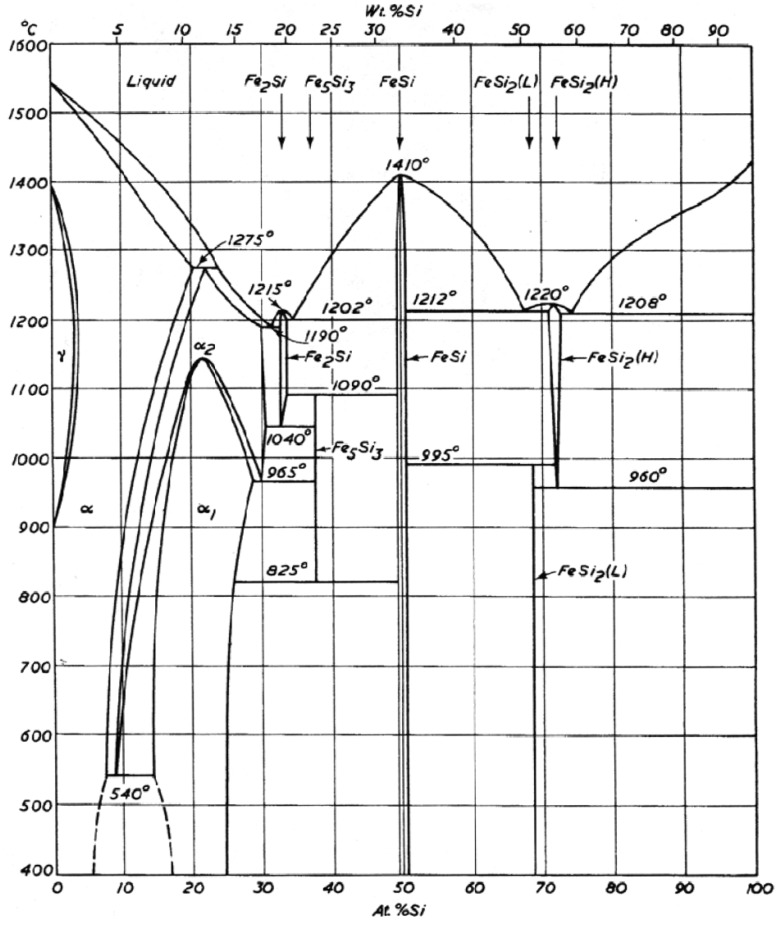
Fe-Si phase diagram. Reproduced from Ref. [12] with permission from Butterworths & Co., London.

The Fe-Si binary phase system provides several compositionally distinct iron silicides that have diversely impressive electronic properties [1]. The well-characterized iron silicides are Fe_3_Si, Fe_5_Si_3_, FeSi (*ε*-FeSi) and FeSi_2_. The silicon-rich FeSi_2_ is known to exist commonly in both *α*- and *β*- forms (Figure 1). *β*-FeSi_2_, which is a semiconductor, has elicited particular interest as it exhibits luminescence at ~1.55 μm. This wavelength is compatible with the current optoelectronic communication technology; especially in the silica optical fiber communication in the infrared region [1]. The search for environmentally and ecologically friendly semiconductors has intensified the interest in iron silicides. This is borne out of the fact that compared to the toxic III–V or II–VI semiconductors made of As, Cd and Se that are typically used in optoelectronic applications, an iron silicide such as *β*-FeSi_2_ is composed of naturally abundant non-toxic elements Si and Fe, which constitute 26% and 5%, respectively, of the earth’s crust [2]. However, the interest in iron silicides is not solely relegated to *β*-FeSi_2_. In fact, the iron-rich silicides, Fe_5_Si_3_ and Fe_3_Si are known to be room temperature ferromagnets with reported Curie (*T*_C_) temperatures of 385 K [3,4,5] and 799 K [6], respectively. Room temperature ferromagnetism has recently been demonstrated in nanodimensional versions of Fe_5_Si_3_ [7,8] and Fe_3_Si [9,10], which opens the possible application of these materials in spintronics [11]. Although Fe_5_Si_3_ is known to be metastable with respect to Fe_3_Si and FeSi below 825 °C [12,13,14], it has been known to exhibit giant magnetoresistance (GMR) properties [3,4,15,16,17], which is the ability of a material to reduce its electrical resistivity in the presence of an external magnetic field [18,19,20,21]. Nanogranular Fe_5_Si_3_ particles formed at a Fe/c-Si interface, on irradiation with 100 MeV swift heavy ions of Fe^+7^, were reported to exhibit a GMR of up to 2400% [16]. This has raised the expectation that Fe_5_Si_3_ might be a room temperature magnetic semiconductor warranting conclusive determination. Magnetic semiconductors are semiconductor materials that possess ferromagnetic properties in the same material due to the mutual interaction between magnetic properties, supported by the *d* electrons of the magnetic ions, and the semiconductor properties, supported by the *s* and *p* electrons, of the semiconductor component of the material [22]. With the *s*, *p*–*d* exchange interaction, one can control the magnetization by the electrical field [23] or control the semiconductor optical characteristics by the magnetic field [24]. Because of the *s*, *p*–*d* exchange interaction, the energies of the *s* and *p* electrons in a magnetic semiconductor become dependent on their spin state, up or down. This could enable the development of nonvolatile digital circuits that retain their logic states even when their power sources are rapidly switched on and off leading to a new type of computer.

The stoichiometric iron silicide, FeSi, is the most thermodynamically stable Fe-Si phase and has been touted to be the first known Kondo insulator with no *f* electrons [25,26,27,28]. Kondo insulators are strongly correlated semiconductors (direct gap) and semi-metals (indirect gap), which exhibit characteristic hybridization of their *f* electrons with conduction bands. The difference in such insulators with conventional semiconductors is in the magnitude of their band gap which exhibit strong temperature dependence due to *e*-*e* interactions. More recently, comparison of high-resolution, angle-resolved photoemission spectroscopy data with *ab initio* band-structure calculations by density functional theory of FeSi has shown that the experimental dispersions in the band gap of FeSi can be quantitatively described by an itinerant behavior when an appropriate self-energy correction has been included [29]. This has led to the assertion that FeSi is not a Kondo insulator [29]. Furthermore, magnetic susceptibility, heat capacity, and field-dependent conductivity studies of the metal-insulator transition in FeSi with Al atoms substituted at the Si sites have revealed that such transitions in FeSi are similar to the ones in classical semiconductors [30]. Hence, even though the status of FeSi as a Kondo insulator is still debated, it suffices to say that the electronic properties of the narrow-gap semiconductor FeSi, with a band gap of 0.55 eV, have interesting fundamental significance and application in light sources or detectors in the near infrared region in optics [31]. To conclude the iron silicide series, as mentioned at the outset, the Si-rich FeSi_2_ phase of *β*-FeSi_2_ is a good semiconducting material with a direct band gap of ~0.85 eV [32,33,34] and has a large optical absorption coefficient [32,33,34] and good physical and chemical stabilities at high temperature [35,36]. It can be used as a silicon-based light emitting diode (LED) [37]. Interestingly, FeSi_2_ exists in two kinds of crystal structures: tetragonal metallic phase (*α*-FeSi_2_) at high temperature and orthorhombic semiconducting phase (*β*-FeSi_2_) at low temperature [33]. The metallic phase, resulting from the existence of vacancies in iron sublattice, occurs within a rather wide range of composition, while the semiconducting phase is stoichiometric. The *α* phase has been known to be stable at elevated temperatures above 950 °C [35,36].

The preceding account validates the significance of the various iron silicides as an important family of binary compounds. In addition to these silicides, silicon-doped iron mixtures named as bcc- and fcc-iron phases are also available from the Fe-Si binary system [12,13,14]. These phases are produced by very low dissolutions of silicon in iron. The dissolution is below 1% and in the range of 1*–*2% for fcc-Fe and bcc-Fe, respectively [12,13,14].

The iron-silicides are typically produced by traditional solid-state reactions [38,39] or by ion beam-induced mixing (IBM) of alternating iron and silicon layers [39]. During solid-state reactions of the (Fe,Si) couple, FeSi has been predicted to form as the first silicide, among the possible iron silicide products, due to its favorable ΔG_v_ (the change in free energy per unit volume or the driving force for nucleation) characteristics [38]. This prediction was corroborated by the formation of FeSi as the first phase in amorphous deposited elemental layers of silicon and iron in a thin-film diffusion couple during thermal annealing [40]. Other iron silicides such as Fe_3_Si, FeSi_2_ and Fe_5_Si_3_ were produced subsequent to the formation of FeSi. The metastable phases, such as Fe_5_Si_3_, formed only when they were easier to nucleate than the more thermodynamically stable phases [40]. One of the means to circumvent this thermodynamic restriction in the formation of iron silicides may be the utilization of vapor and solution-state reactions or variants of solid-state reactions. In such reactions, the ΔG_v_ situations can be tailored such that less thermodynamically-favored iron silicides can be produced by introducing additional interfacial free energy (γ) during the formation of the products.

**Figure 2:**
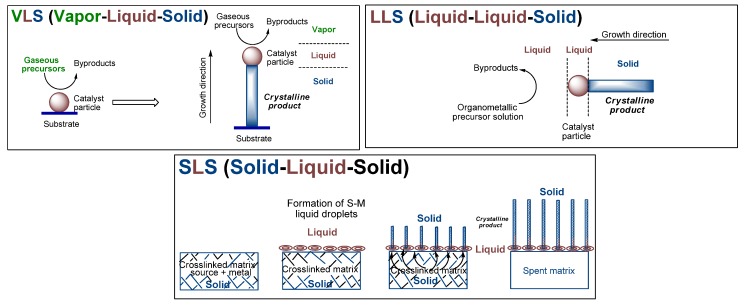
Schematic representations of the Vapor-Liquid-Solid (VLS), Liquid-Liquid-Solid (LLS) and Solid-Liquid-Solid (SLS) mechanisms. VLS and LLS mechanisms, adapted from Ref. [45] with permission from The American Association for Advancement of Science, and SLS mechanism, adapted from Ref. [47] with permission from Elsevier.

Reactions of organometallic compounds that contain both Fe and Si provide favorable routes whose energetics can be tailored to access the various iron silicides. The reactions can be performed in gas, liquid or solid phases. It offers several advantages including initiation of the reactions under milder conditions, and improved control over composition, structure and final form of the expected material [41,42]. The attending mechanisms for such reactions encompass broadly the commonly known vapor-liquid-solid (VLS) [43,44], liquid(solution)-liquid-solid (LLS) [45,46] or solid-liquid-solid (SLS) [47,48] mechanisms (Figure 2). The commonality in such reactions is the interaction of a volatile reactant component with a solid reactant component which typically results either in the dissolution of the volatile component in the solid component to form mixed systems, as in the CVD processes, or of strong surface interactions between the two components, as in epitaxy processes, both of which results in the precipitation of products. In particular, the progress of dissolution results in supersaturation, nucleation and precipitation of the lowest melting phase, typically in the form of a crystalline solid product. A notable difference among the mechanisms is that in both VLS and LLS processes, the dissociation of the reactants occurs in a large reaction volume, such as in a CVD chamber or a reaction vessel, with the attending mediation of gas or liquid molecules. However, in the SLS mechanism, such dissociation occurs within the confines of a networked matrix obtained by the crosslinking of a precursor which contain both the organic and the metal moieties within the polymeric backbone. Thus, SLS reactions typically utilize single source organometallic precursors (SSPs). In the VLS and LLS processes the precursors are generally transported to the site of the catalyst by either a gas or a liquid, respectively. Hence, the nature of the delivered reactive precursor composition depends also on the outcome of the interaction between the precursors with the carrier gas or the liquid in accordance with gas and fluid dynamics. This scenario does not exist in the SLS-type reactions, since the reactions occur in the confines of the solid-state matrix. As a result, in VLS and LLS-type reactions the precursor compositions tend to approach the thermodynamic equilibrium, whereas in the SLS-type reactions such equilibration may not be achieved due to the spatial constraints imposed on the reactant precursors in the crosslinked networked matrix. This can lead to the formation of unique phases in SLS-type reactions that are typically not accessible by VLS or LLS-type reactions. This is especially possible by the custom tailoring of the spatial interactions in the system by the selective spatial placement of the reactive components in the precursor which leads to preferential sequestration and consequent segregation of desired components. Thus, all of these reaction mechanisms have their own unique advantages.

As apparent from the preceding discussion, the organometallic compounds usable in the synthesis of iron silicides do not have to be small molecules or compounds containing Fe and Si in spite of their demonstrated utility (*vide infra*). In fact, the development of organometallic polymer precursors that can be controllably pyrolyzed to iron silicides has provided a new class of macromolecular preceramic organometallic entities [49,50,51,52,53]. The polymer precursor route, prevalent in the SLS-type reactions, offers several noteworthy advantages, such as the provision of a large palette of means to manipulate the structure and properties of the formed products due to the diversity in chemical compositions of organometallic macromolecules and the, previously discussed, controlled placement of the Fe and Si atoms distributed along the macromolecular chains and segregated by the organic moieties. Upon the pyrolysis of the polymers, nanoclusters with interconnection may be generated with mesoporous morphologies. In addition, the unique processing characteristics of the precursor polymers may enable the fabrication of bulk ceramic bodies of complex shapes [54], which should facilitate the easy access of nanorealms of the iron-silicon phases. In all of the foregoing organometallic reactions involving either monomeric or polymeric species that produce the various iron silicides, iron can be assumed to play the role of the catalyst and silicon the role of the nanostructure-initiating component, since silicon is known to dissolve well in iron while the case of the reverse dissolution is not facile, as the solubility of Fe in Si has been established to be extremely small (less than 0.005 at %) even at temperatures as high as 1300 °C [55].

This review will describe the various organometallic reactions that lead to nanomaterials of iron silicides. The review is formatted in the following fashion. In the first section, the thermodynamic stabilities of the various iron silicides will be discussed as a predictor for the propensity of the precursor materials to form the various iron silicides. This will be followed by the examples of various nanoscale iron silicides that have been formed from organometallic species, the products being listed in the order of their iron-richness; the iron-rich Fe_3_Si and Fe_5_Si_3_ examples being discussed initially, followed by the examples of the stoichiometric FeSi and, finally, of the silicon-rich FeSi_2_ examples.

## 2. Results and Discussion

### 2.1. Thermodynamic stabilities of the various iron silicide phases

In perusing the existing examples of known iron silicides, one may be tempted to predict that the prevalence or the paucity of a particular iron silicide is an outcome of its thermodynamic stability, which happens to be the case to a large extent. The first report on the thermodynamic properties of the iron silicide systems by Korber and Oelsen appeared in 1936 [56]*.* The ΔH°_298_ values were reported for the stable iron silicides Fe_3_Si, FeSi and FeSi_2_ and were −20.1, −40.2 and −23.6 kJ/mol, respectively. More recently, an authoritative review [57] by Schlesinger has suggested that the best choice of experimental technique for determining the ΔH°_298_ values for these silicides is metal solution calorimetry. In accordance, the ΔH°_298_ results of Jounel *et al.* [58] for FeSi of −39.3 kJ/mol, of Sommer [59] for Fe_3_Si of −25.8 kJ/mol, and of Gorelkin and Mikhailov [60] of −30.6 kJ/mol for FeSi_2_ were recommended. Thus, the relative order of stability and ease of formation follows the series: FeSi > FeSi > Fe_3_Si (−39.3 > −30.6 > −25.8 kJ/mol). Even though the ΔH°_298_ value of the metastable Fe_5_Si_3_ was not available in this report, it can be safely assumed that its metastability would place its thermodynamics of formation at the opposite end with respect to FeSi in the series.

In a contrasting report on thermodynamic stabilities of iron silicides by Krausze *et al.*, [61] the Δ_f_H°_298_ (kJ/mol) of the iron silicides appear in the following series: Fe_5_Si_3_ > Fe_3_Si > FeSi_2_ > FeSi: (−244.9 > −94.2 > −81.2 > −73.7); thereby seeming to suggest that an iron silicide with a greater amount of iron forms easier. However, as apparent in most reports on iron silicides, the order reported by Schlesinger seems to be the chosen one.

### 2.2. Organometallic reactions that produce nanomaterials of Fe_3_Si

There are only two reports of organometallic reactions that lead to the production of nanoscale Fe_3_Si products. The two reactions involve pyrolysis of solid matrixes that are produced from oligomeric organometallic precursors; thus, belonging to the SLS-type of reactions.

**Figure 3:**
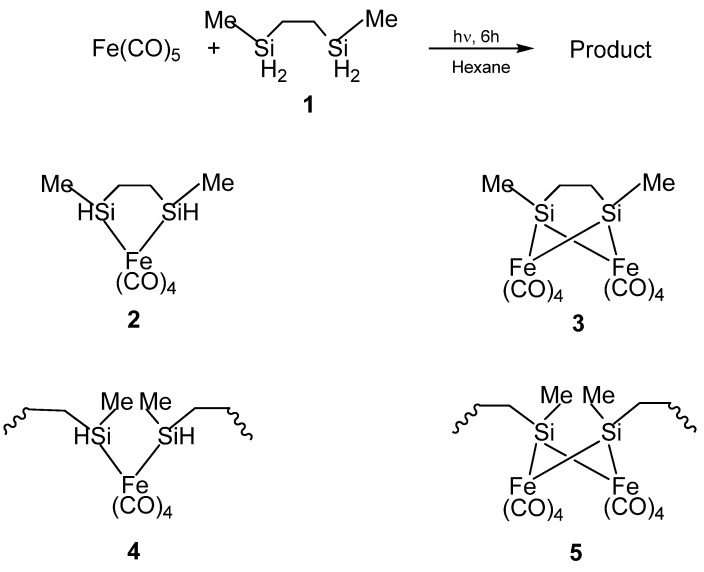
(Top) The reaction to form the organometallic product. (Bottom) The possible structures of the product formed from the reaction, with **2** and **4** being the probably formed ones based on elemental analysis and IR spectroscopy. From Ref. [62], Adapted by permission of The Royal Society of Chemistry.

In the first report by Corriu *et al.*, the organosilicon polymeric materials that served as the precursors for nanoscale Fe_3_Si were synthesized by the reactions of 2,5-disilahexane with iron carbonyl (Fe(CO)_5_) [62]. The structures of these polymeric materials were obtained by NMR and IR spectroscopic characterizations which indicated the presence of mixed oligomeric and molecular species. The initial reaction of the Si-H bonds with Fe(CO)_5_ was found to proceed by both intra- and inter-molecular reactions resulting in the formation of Si-M bonds in monomeric or oligomeric structures. The result was quite unexpected because the four Si-H moieties of the 2,5-disilahexane were expected to form four Si-M bonds thereby resulting in the bridging of the 2,5-disilahexane unit by the iron carbonyl moieties which would have produced an insoluble polymer network (such as the Structure **5** in Figure 3), or, at least, a very complicated mixture of oligomers [63]. However, the results suggested that since the reactions were carried out under mild conditions (0.1 M, room temperature, 24 h), the reaction of the Si-H bond was limited. Therefore, it was possible to form iron complexes that still contained unreacted Si-H functions. Thus, in the product, the Si atoms were assumed to be linked mainly to only one transition metal center based on ^29^Si NMR spectroscopy studies. The authors confirmed that the major compound (Structure **4** in Figure 3) present in the product contained an average of one Si-H bond per Si atom by elemental analysis and IR spectroscopy.

**Figure 4:**
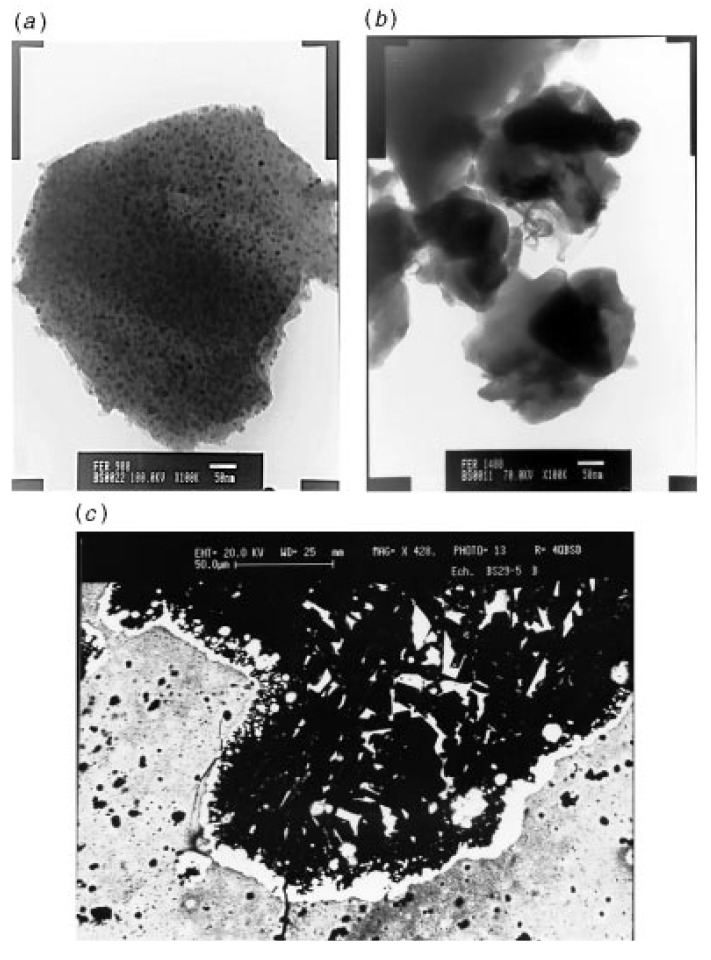
(a) TEM photograph of a 1000 °C sample, (b) TEM photograph of a spherical metallic particle, and (c) BEI photograph of an interfacial melting zone observed in a 1400 °C sample. From Ref. [62], Reproduced by permission of The Royal Society of Chemistry.

The pyrolysis of crosslinked **4** under argon to a temperature of 1000 °C was observed to yield a product with multiphase ceramics in high yields. A portion of the CO ligands present in **4** was found to be incorporated in the product during the thermal decomposition. The materials were characterized by X-ray, Raman, TEM and magnetic susceptibility analyses. In terms of iron silicides, crystalline Fe_3_Si particles that were 10 nm in size were mainly detected in the product. A fraction of amorphous oxide or oxycarbide phases was also present in the product which was consistent with its observed oxygen level (Figure 4). A carbothermal reduction of this product between 1200 °C and 1400 °C was found to result in the elimination of CO and SiO and the modification of the ceramic phases. The carbothermal reduction also resulted in the formation of SiC and FeSi. Melting of the Fe_3_Si phase was found to lead to the formation of spherical metallic particles (Panel (b) in Figure 4).

**Figure 5:**
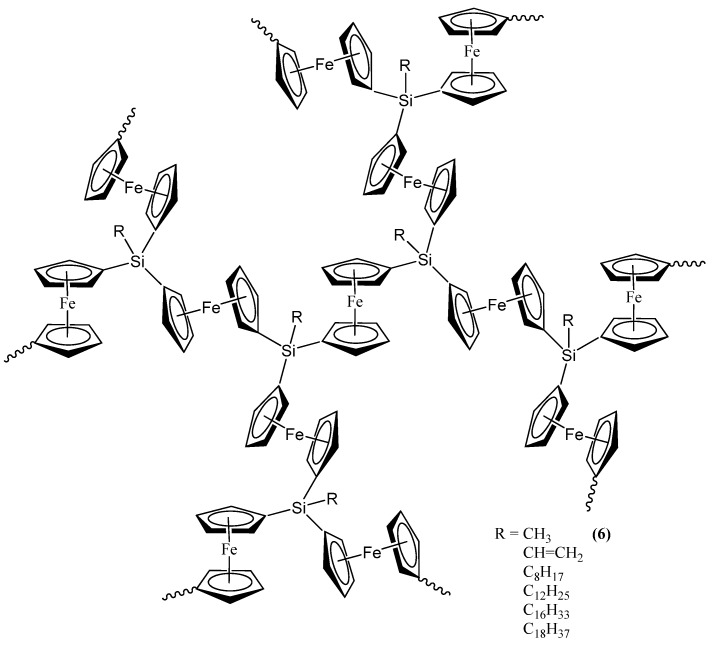
The methyl-containing hyperbranched poly[1,1’-ferrocenylene(methyl)silyne], **6**, used in the production of the Fe_3_Si-containing product **7A**. Shown, also, are the variants of **6** that were utilized in the study. Adapted from Ref. [65] with permission from American Chemical Society.

**Figure 6:**
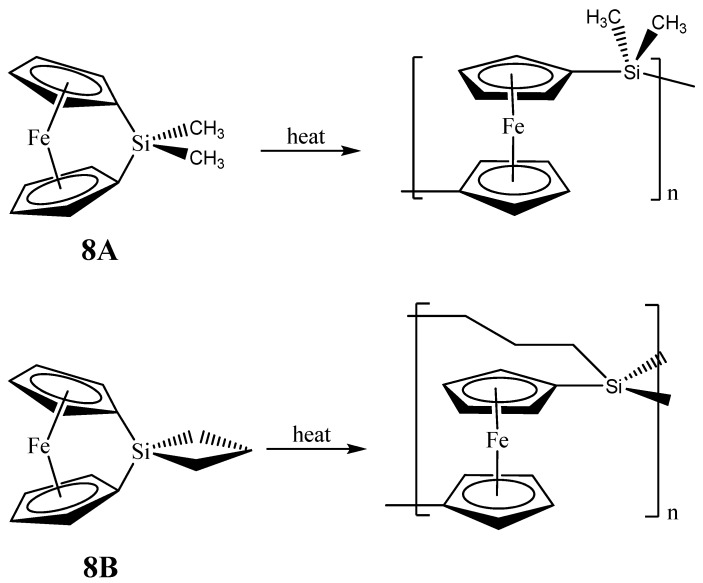
Ring opening polymerizations (ROP) of 1,1-ferrocenylene(dimethyl)silylene (**8A**) and spirocyclic[1]-ferrocenophane (**8B**) that produce the respective crosslinked networks. Adapted from Ref. [68] with permission from American Chemical Society.

The second set of reports of the production of Fe_3_Si nanoparticles from an organometallic system by Tang *et al.* results from the pyrolysis of hyperbranched poly[1,1’-ferrocenylene(methyl)silyne], **6** (Figure 5) [64,65,66]. As in the Corriu example, the Fe_3_Si-yielding precursor is a crosslinked hyperbranched material that is subjected to an SLS-type reaction. Depending on the atmosphere of pyrolysis, which was either nitrogen or argon, **6** was found to convert to **7N** and **7A**, respectively, in ~48*–*62% yields at high temperatures (1000*–*1200 °C). Thus, the ceramization yields of **6** were found to be higher than that of its linear counterpart poly[1,1-ferrocenylene(dimethyl)silylene] (**8A**) (~36%) (Figure 6) [67], revealing that the hyperbranched polymer was superior to the linear one as a ceramic precursor. In this regard, it is apt to note that in the hyperbranched polymer **6**, each of the silicon atoms is surrounded on an average by 3/2 or 1.5 ferrocenyl moieties. Thus, the theoretical iron content is higher than of its linear counterpart **8A**, in which the silicon is linked to only one ferrocenyl moiety in the monomer. Hence, it is not a surprise that **6** has a propensity to form the most iron-rich silicide Fe_3_Si in comparison to Fe_5_Si_3_ formed by **8B**, the spirocyclic counterpart of **8A** (*vide infra*). The nanocrystals in the ceramics formed from **6** in either nitrogen or argon were found to be of sizes in the range of 41–117 nm. The nanocrystals in **7N** and **7A** were characterized by SEM, XPS, EDX, XRD, and SQUID. It was found that the ceramics were electrically conductive and possessed a mesoporous architecture constructed of tortuously interconnected nanoclusters. The iron contents of **7N** and **7A** estimated by EDX were 36–43%, which was much higher (by ~11%) relative to the ceramic **9** prepared from the linear precursor **8A** or **8B**. Interestingly, the nanocrystals in **7N** were found to be mainly *α*-Fe_2_O_3_ whereas those in **7A** were mainly Fe_3_Si. This type of differentiation in the products as a function of the reactant atmosphere was also observed in the case of the products of **8B** (*vide infra*) and is a prime example of the opportunity that SLS-type of reactions provides in the selectivity of the reaction products. The product **7A,** containing Fe_3_Si nanocrystals, was found to exhibit a high-saturation magnetization (*M_s_* ~49 emu/g) and near-zero remanence and coercivity, when magnetized by an external magnetic field at room temperature. Thus, **7A** appeared to be an excellent soft ferromagnetic material with an extremely low hysteresis loss. The authors also made several variants of **6**, by changing the alkyl group on the silicon atom from methyl to *n*-dodecyl. It was found that the ceramic yield increased with a decrease in the alkyl chain length of the hyperbranched poly[1,1’-ferrocenylene(*n*-alkyl)silyne] precursors, with the highest yield being obtained with the polymer containing the smallest alkyl (*i.e.,* methyl) group.

### 2.3. Organometallic reactions that produce nanomaterials of Fe_5_Si_3_

As with the Fe_3_Si case, there are only two examples of organometallic reactions that lead to the production of nanoscale Fe_5_Si_3_ materials. In the first report, Manners and Ozin reported the production of magnetically tunable ceramics from the pyrolysis of cross-linked polyferrocenylsilane networks obtained from the linear spirocyclic[1]-ferrocenophane **8B** (Figure 6), alluded in the previous section [68]*.* The SLS-type pyrolysis reaction occurred in the solid matrix that was produced from **8B** eventually leading to the products. The cross-linked polyferrocenylsilane network was derived from the ring-opening polymerization (ROP) of the **8B** precursor. The pyrolysis product of crosslinked **8B** under nitrogen to 1000 °C was found to yield shaped macroscopic magnetic ceramics **10N** consisting of *α*-Fe (bcc-Fe) nanoparticles, a non silicide product from the Fe-Si phase diagram, embedded in a SiC/C/Si_3_N_4_ matrix in greater than 90% yield. Incidentally, the production of bcc-Fe nanoparticles was also observed in the second example of Fe_5_Si_3_ nanoparticle production reported by Kolel-Veetil *et al*. (*vide infra*) [8]. Variation of the pyrolysis temperature and time permitted control over the nucleation and growth of bcc-Fe particles, which ranged in size from around 15 to 700 Å, and the crystallization of the surrounding matrix, as typical of the SLS-type mechanism. The ceramics contained smaller bcc-Fe particles when prepared at temperatures lower than 900 °C and displayed superparamagnetic behavior, whereas the materials prepared at 1000 °C contained larger bcc-Fe particles that were ferromagnetic. Such flexibility in the control of the magnetic property may be useful for particular materials applications. Interestingly, the composition of the ceramic was found to alter when the pyrolysis atmosphere was changed to argon. The ceramic **10A** obtained from such a pyrolysis was found to contain Fe_5_Si_3_ nanoparticles of sizes 48 ± 2 Å.

 There are a couple of interesting aspects to this study. Firstly, the identity of the products remained unaltered in N_2_ when the pyrolysis was performed to a greater duration at different temperatures. Thus, only the size of the bcc-Fe nanoparticle was observed to increase substantially (from 10 nm to 63 nm) when the pyrolysis was carried out to a longer duration and at higher temperatures, with the change in duration from 2 h to 24 h and the temperature ranging from 650 °C to 1000 °C. Secondly, and more importantly, a change in the reaction environment from N_2_ to argon was found to alter the identity of the nanoparticle product from bcc-Fe to Fe_5_Si_3_. Therefore, it is interesting that in this SLS-type reaction a change in the reaction environment causes a change in the nature of the product. A reason for this could be that the substitution of nitrogen by argon precluded a nitrogen-mediated pathway for the production of bcc-Fe nanoparticles along with the formation of *α*-Si_3_N_4_, Fe_4_N and Fe_2_N as observed in that case. Even though in argon, as in N_2_, only bcc-Fe nanoparticles were formed up to 600 °C, above this temperature the bcc-Fe nanoparticles reacted with their surroundings to form Fe_5_Si_3_ nanoparticles. The report, unfortunately, did not describe the magnetic properties of the Fe_5_Si_3_ nanoparticles. However, the magnetic properties of the bcc-Fe samples were reported. The saturation magnetization for bcc-Fe nanoparticles contained in the 650 °C and 2 h sample was 7 emu g^-1^ at 300 K and 10 emu g^-1^ at 100 K; higher values were observed for the 850 °C and 2 h sample (32 emu g^-1^ at 300 K and 36 emu g^-1^ at 100 K) as expected due to the presence of more iron particles. These samples showed no hysteresis at 100 or 300 K, and the magnetic saturation was found to be gradual, consistent with the behavior of superparamagnetic particles.

The magnetization of the larger bcc-Fe particles was reported to rapidly reach saturation when heated at 1000 °C for 2 h. The ceramic was shown to possess a saturation magnetization of 44 emu g^-1^ and coercive field of 10 G and to display room-temperature hysteresis and a small remnant magnetization (1 emu g^-1^) consistent with a soft ferromagnet. In essence, the bcc-Fe particles had become large enough (larger than a single Weiss domain) to display ferromagnetism. In this regard, the maximum size of a single domain for a perfectly spherical Fe particle is calculated to be 14 nm [69]. SQUID magnetometry studies performed in the study demonstrated a transition from small superparamagnetic bcc-Fe particles to larger ferromagnetic bcc-Fe particles at about 900 °C. Thus, by varying the pyrolysis temperature, the bcc-Fe particle size and magnetic properties of the resulting ceramics was found to be tunable. This processing flexibility may be advantageous for particular materials applications.

**Figure 7:**
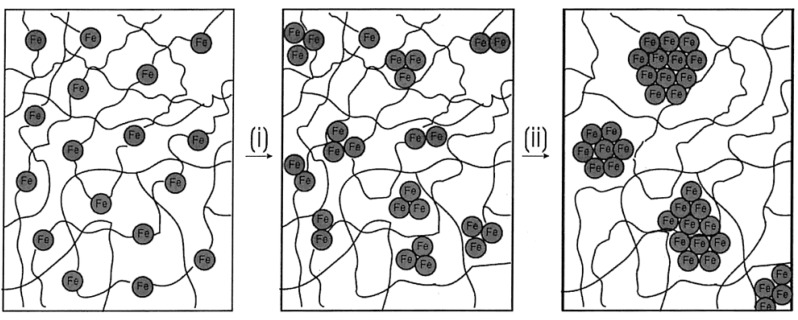
Representation of a nucleation and growth model that illustrates the genesis of the magnetic ceramic **10N** from (i) the iron atom release from polymer **8B** followed by (ii) nucleation and growth of iron nanoparticles. Reproduced from Ref. [68] with permission from American Chemical Society.

A model was also presented by the authors for showing how the genesis of magnetic ceramic from cross-linked polyferrocenylsilane depended on pyrolysis conditions (Figure 7). The network of cross-linked polymer of **8B** was proposed to expand during the initial stages of heating. Further heating resulted in the “release” of Fe atoms from the ferrocene moieties. The disappearance of ferrocene units by 650 °C was supported by UV-vis and near-IR spectroscopy evidences. Further dismantling of the polymer matrix was expected to occur concomitantly during the nucleation and growth of the bcc-Fe particles. At 700 °C, the bcc-Fe particles were expected to catalyze the formation of graphitic ribbons in the ceramic; followed by the simultaneous growth in size of both species with temperature. The bcc-Fe particles were observed to form with a bimodal particle size distribution possibly due to the formation of larger particles at grain boundaries and defect sites as a result of faster intergrain iron atom diffusion. N1s XPS studies of the ceramic were found to exhibit the incorporation of nitrogen at 700 °C, which was later incorporated into crystalline silicon and iron nitrides as observed by powder XRD. A small amount of Fe_4_N was observed to form at 850 °C and to be localized at the ceramic surface. This “competition” for Fe atoms was proposed to cause a decrease in bcc-Fe particle size at 900 °C, which was followed by the crystallization of *α*-Si_3_N_4_ and a small amount of Fe_2_N at 950 °C. Magnetic measurements showed that as the bcc-Fe particles became larger, they underwent the expected transition from superparamagnetic to ferromagnetic behavior. Particles formed below 900 °C contained bcc-Fe particles that were smaller than a single Weiss domain and were found to behave as superparamagnets, whereas larger particles prepared at 1000 °C conferred ferromagnetic properties to the ceramic. Although the authors did not elaborate on the mechanism of formation of the Fe_5_Si_3_ nanoparticles, it is tempting to conjecture what effect does the presence or absence of nitrogen (when argon is used in the pyrolysis) has on the dissolution of silicon in iron after the “release” of the iron particles and also on the local availability of silicon in the segregated environments present in a SLS-type system. It is possible that the iron and silicon reactants are spatially separated to an extent that in the presence of nitrogen the rate of nitrogen dissolution exceeds the rate of silicon dissolution in iron thereby yielding the bcc-Fe particles. Conversely, in the absence of nitrogen this spatial restriction becomes inconsequential and the formation of Fe_5_Si_3_ nanoparticles results.

**Figure 8:**
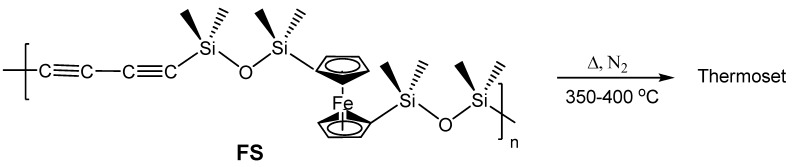
The diacetylene-containing ferrocenylsiloxane polymer **FS** and its conversion into a thermoset by heating. Adapted from Ref. [8] with permission from American Chemical Society.

The only other example of formation of Fe_5_Si_3_ nanoparticles from an organometallic species is from the more recent report by Kolel-Veetil *et al.* wherein the pyrolysis of a networked system obtained from a diacetylene-containing ferrocenylsiloxane polymer (**FS**) (Figure 8) produced bcc-Fe nanoparticles at slower rates of pyrolysis and Fe_5_Si_3_ nanoparticles at higher rates of pyrolysis [8]. The pyrolysis reactions which belong to the SLS-type of reactions, as in the report by Manners and Ozin [68], were carried out only in a nitrogen atmosphere and are novel in that the selectivity in the products occurred merely by the change in the kinetics of the pyrolysis as opposed to a change in the reaction gas in the example by Manners and Ozin. The polymer precursor was formed by the reaction of dilithiated diacetylene and dilithiated ferrocene with dichlorotetramethyldisiloxane. The dilithiated diacetylene was produced by the conversion of hexachlorobutadiene by reaction with *n*-BuLi as reported by Barton *et al.* [70]*.* The precursor polymer **FS**, which formed in high yields, contained a Si mole fraction of 0.8 in the polymer making it a silicon-rich system. Based only on the Fe-Si phase diagram, such a system was expected to form either the thermodynamically-favored stoichiometric FeSi or the silicon-rich FeSi_2_ on pyrolysis when considered as a purely binary system. However, understandably, the presence of the additional elements C, O and H was found to exert additional influence on the nature of the pyrolysis products. The products were obtained by the pyrolysis of the thermoset of **FS** formed by treatment of the polymer at 400 °C for 2 h. Further treatment of this thermoset in nitrogen at either 1 °C/min or 10 °C/min resulted in the formation of either Fe_5_Si_3_ nanoparticles or bcc-Fe nanoparticles, respectively. The x-ray diffraction (XRD) and energy dispersive spectra (EDS) of both products showed only the presence of C as an additional element in the mixture. Hence, the reactant systems can be considered as ternary systems comprising Fe, Si and C. TEM evaluation of the products revealed bimodal size distribution in both products and the EDS spectra of the products showed the expected concentration of Fe in the bcc-Fe nanoparticles and the concentration of Fe and Si in the Fe_5_Si_3_ nanoparticles, respectively. Even though the majority of the Fe_5_Si_3_ particles existed in the 10–15 nm range, ~20% of the larger Fe_5_Si_3_ nanoparticles present were as large as 40 nm. The bcc-Fe nanoparticles existed in the bimodal distribution size range of 10–40 nm and 75–100 nm. Silicon was found to be highly dispersed in the matrix containing the bcc-Fe nanoparticles. However, in the Fe_5_Si_3_ nanoparticles-containing composition additional Si was found to be sparsely dispersed in the matrix.

**Figure 9:**
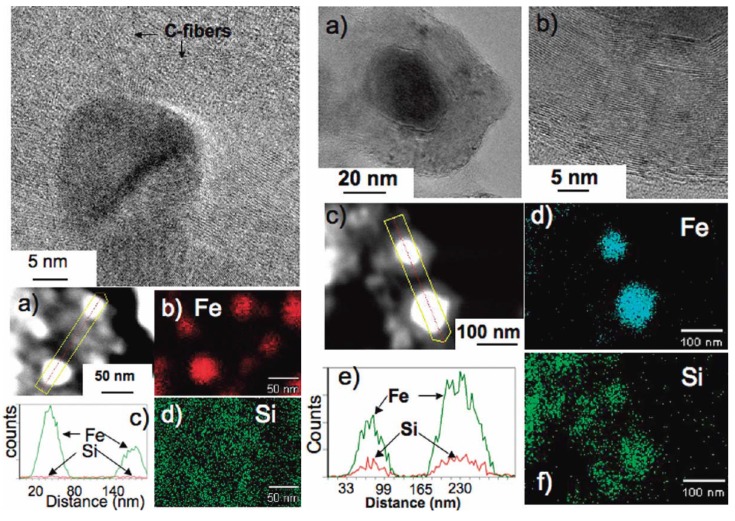
(Left) A HRTEM image showing bcc-Fe nanoparticles with protruding carbon fibers, (a) a HAADF image of two bcc-Fe particles encapsulated by carbon, (c) an EDS line scan across the two particles showing the presence of Fe, and (b) and (d) fine probe EDS maps for Fe and Si (present dispersed in the matrix), respectively. (Right) (a) A HRTEM image showing a Fe_5_Si_3_ particle encapsulated by a carbon nanocapsule, (b) a high magnification of a portion of the carbon nanocapsule showing the (0002) lattice fringes, (c) a HAADF image of two Fe_5_Si_3_ particles encapsulated by carbon, (e) an EDS line scan across the two particles showing the presence of Fe and Si, and (d) and (f) fine probe EDS maps for Fe and Si, respectively. Reproduced from [8] with permission from American Chemical Society.

**Scheme 1:**
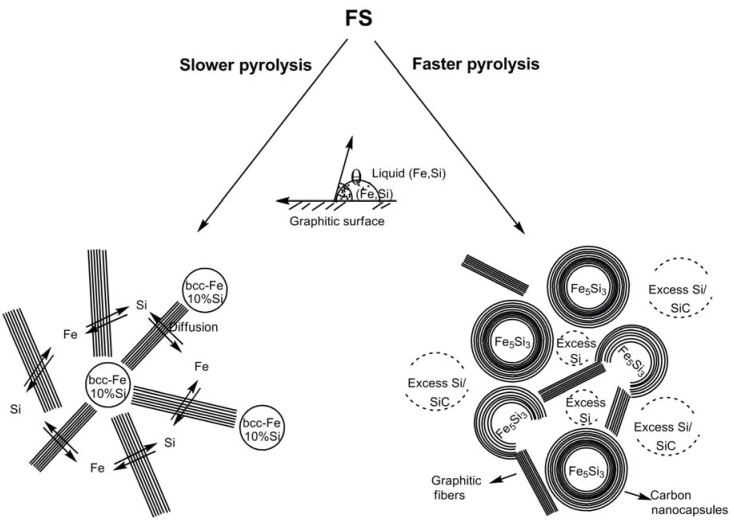
Representation of pathways to form nanoparticles of silicon-doped bcc-Fe and Fe_5_Si_3_, respectively, at slower and faster pyrolysis rates. Adapted from Ref. [8] with permission from American Chemical Society.

It was of interest to determine the origin of the differentiation in the products at the slower and faster pyrolysis rates. High resolution TEM (HRTEM) studies of the products revealed that in the faster pyrolysis product the carbon component existed as nanocapsules that encapsulated the Fe_5_Si_3_ nanoparticles, whereas in the slower pyrolysis product the carbon component was seen in the form of graphitic ribbons and other shaped-moieties that protruded from the periphery of the bcc-Fe nanoparticles (Figure 9). The divergence in the reaction pathway and formation of distinct Fe species above 800–900 °C during pyrolysis at the two rates were believed to be an outcome of the different interfacial interactions that the evolving silicon-dissolved iron species have with the carbon component as a consequence of the variation in the catalytic abilities of the two Fe species (Scheme 1) [71,72,73]. During faster pyrolysis, the evolving Fe entities with a greater amount of dissolved Si were proposed to preferentially etch the surrounding graphitic structure more than catalyze its linear growth, resulting in its conversion to carbon nanocapsules. However, at slower pyrolysis, Fe catalyst entities, with a lower amount of dissolved Si, were proposed to preferentially catalyze the formation of linear graphitic nanofibers. Similar major modifications in graphitic nanofiber growth characteristics due to the presence of a metal or a nonmetal adatom such as Si in group 3d metal catalysts (Fe, Co and Ni) have been reported and are believed to be due to different wetting properties [*γ*, *f*(*θ*)] of the metal catalysts with the developing graphitic units [71,72,73]. Such adatoms manifest as perturbations to the gas-metal and metal-solid carbon interfaces resulting in a change in the catalytic efficiency of the activity of the carrier catalyst. Under such a scenario, there was presumed to be a competition between the etching rate of the evolving graphitic moiety and the catalytic efficiency of the metal catalyst to produce more of the graphitic entity. It has been postulated that when the etching rate dominates, encapsulation of the catalyst by the graphitic moiety occurs, and conversely, when the catalytic efficiency dominates, the formation of linear graphitic nanostructures takes place [73].

The authors performed thermodynamic calculations of the Gibbs free energy in the 800*–*900 °C range, where divergence of the reaction pathway occurred, as a function of mole fraction of Si to predict the different phases of the Fe-Si system. The results clearly indicated that there was a considerable driving force for the formation of Fe_5_Si_3_ from liquid Fe-Si below 50% Si. The Fe_5_Si_3_ phase was found to be in equilibrium with the bcc-Fe(Si) phase. A common tangent between bcc-Fe(Si) and Fe_5_Si_3_ was observed at 900 °C, suggesting that bcc-Fe(Si) with 30% Si can be in equilibrium with Fe_5_Si_3_. Even though FeSi was also favored to be formed at this temperature, it was postulated that the presence of the extraneous carbon component shifted the Si mole fraction into the 0.3*–*0.4 range possibly due to the separation of the Fe and Si reactants, thereby limiting the availability of Si for reaction with Fe and consequently thwarting the formation of FeSi.

In comparing the two examples of Manners and Ozin, and Kolel-Veetil *et al.* for the formation of Fe_5_Si_3_ nanoparticles, it is interesting that in the report by Kolel-Veetil *et al.* the formation of Fe_5_Si_3_ occurs from a crosslinked polymer containing Fe, Si and C, such as in Manners and Ozin’s case, in spite of the presence of nitrogen. By way of conjecture, it may be postulated that the type of the tight carbon crosslinks formed from diacetylene groups in the Kolel-Veetil *et al.*’s case perhaps prevented the access of iron by nitrogen in such networks, as evident from the lack of formation of any Fe_2_N or other iron nitrides, whereas such a scenario does not exist in the case of the less tightly-bound networks formed from the spirocyclic group in the spirocyclic[1]-ferrocenophane **8B** in the example of Manners and Ozin. Thus, it provides yet another example of the tailorability of interactions of reactive components in crosslinked networks produced in SLS-type reactions.

The saturation moment of the sample with the Fe_5_Si_3_ nanoparticles was determined to be ~7 emu/cm^3^, while the corresponding value for the silicon-doped bcc-Fe nanoparticle-containing sample was found to be ~16 emu/cm^3^. The small values were consistent with the large volume of the carbon matrix compared to the magnetic particles. The Curie temperature (*T*_C_) determination of the products by vibrating sample magnetometer measurements was found to yield a *T_C_* value of 375 K for the Fe_5_Si_3_-containing product, which was close to the reported *T*_C_ of Fe_5_Si_3_ of 385 K [16]. The *T*_C_ of the silicon-doped bcc-Fe nanoparticle-containing sample was estimated to be 1043 K.

The magnetoresistance (MR) of both samples were measured and reported between 0 and 6 T at temperatures of 100, 200, and 300 K. At room temperature, the Fe_5_Si_3_ nanoparticle-containing product was found to exhibit a negative MR, while the silicon-doped bcc-Fe nanoparticle-containing product did not display a MR. A negative MR was also observed for the Fe_5_Si_3_ sample at 200 and 100 K, while positive MR was observed for the silicon-doped bcc-Fe sample at these temperatures. The MR of the Fe_5_Si_3_-containing product was determined to be −0.6, −0.7, and −0.9% at 300, 200, and 100 K, respectively. The corresponding values for the silicon-doped bcc-Fe-containing product was determined to be 0.0, +0.5, and +0.9%. Thus, the highest MR observed for both samples was around 1%.

The authors suggested that the MR of the Fe_5_Si_3_ nanoparticle-containing sample was far less than the 2400% GMR previously observed [16] for the Fe_5_Si_3_ formed at the Fe/c-Si boundary because of weak coupling between the Fe_5_Si_3_ nanoparticles, since the magnetic Fe_5_Si_3_ nanoparticles were embedded in a carbon matrix of considerable volume with the particles rather distant from one another. Since positive and negative MR of small magnitudes can be caused or influenced by several factors, including electronic band structure, weak localization, electron-electron interactions, and spin-orbit scattering and since these phenomena were dependent on the microscopic properties of materials, it was reported that due to the lack of thorough chemical and structural studies of the interfacial regions of the samples, the source of the MR could not be determined. In addition, it was reported that any quantitative treatment of the MR would require a more detailed analysis of the transport properties of the carbon matrix.

**Figure 10:**
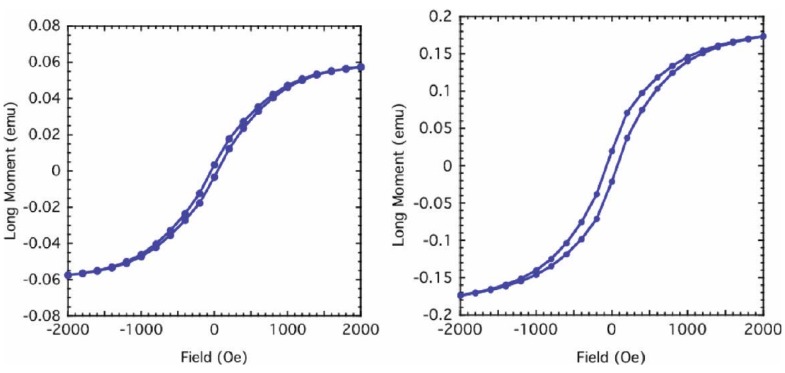
The magnetization of the Fe_5_Si_3_ nanoparticle-containing product (left) and the silicon-doped bcc-Fe nanoparticle-containing product (right) as a function of the magnetic field; the hysteresis loop being depicted. Reproduced from Ref. [8] with permission from American Chemical Society.

Kolel-Veetil *et al.* further reported the M-H curves of both samples which exhibited hysteresis at room temperature (Figure 10). The existence of hysteresis suggested the presence of magnetically coupled nanoparticles in the samples [74]. The coercive field of the Fe_5_Si_3_-containing sample was found to be around 50 Oe. In reported studies of the magnetic properties of nanocrystalline Fe_1-*x*_Si*_x_* alloys (0.15 < *x* < 0.34), the lowest coercive field of 2.9 Oe was found for a stoichiometry of Fe_0.67_Si_0.33_, which is the metastable Fe_2_Si (B2) phase [6,75,76]. On annealing this metastable phase above 800 K, a decomposition into the neighboring stoichiometric Fe_5_Si_3_ and Fe_3_Si phases was induced, and the coercive field was observed to be enhanced by nearly an order of magnitude (from 2.9 to 20.8 Oe). This increase in coercivity was attributed to the formation of the hexagonal Fe_5_Si_3_ phase [4,75,76]. Furthermore, a relationship between the coercive field and film thickness has been reported in in-plane hysteresis loops of sputtered polycrystalline films of Fe_5_Si_3_ [15,16,17]. For a 65 nm thick film, the coercive field at room temperature was found to be about 200 Oe. However, on increasing the film thickness to 500 nm, the room temperature coercive field was found to decrease to 97 Oe. In light of such data, the authors opined that the coercivity of the Fe_5_Si_3_ nanoparticle-containing sample appeared to be in the range of reported coercivity values of Fe_5_Si_3_-containing compositions and films. In comparison, the coercive field of the silicon-doped bcc-Fe nanoparticle-containing sample was found to be around 200 Oe, indicating its existence as a moderately hard magnetic system.

### 2.4. Organometallic reactions that produce nanomaterials of FeSi

**Figure 11:**
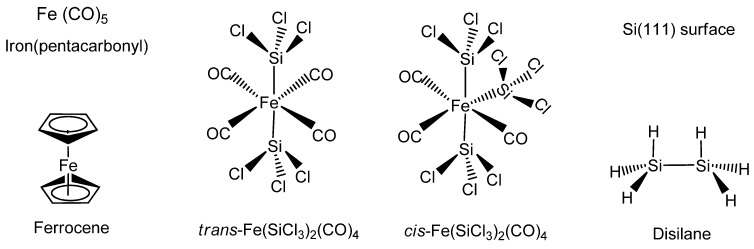
Structures of the typical organometallics used in the formation of FeSi, such as Fe(CO)_5_ (Iron pentacarbonyl), Fe(C_5_H_5_)_2_ (Ferrocene), *cis*-Fe(SiCl_3_)_2_(CO)_4_, *trans*-Fe(SiCl_3_)_2_(CO)_4_, Si_2_H_6_ (Disilane) and Si(111) surface.

The organometallic synthesis of the stoichiometric iron silicide FeSi has been achieved mainly by using the iron complexes Fe(CO)_5_ or Fe(C_5_H_5_)_2_ as the iron precursor (Figure 11). The silicon component has been derived from either the volatile precursor Si_2_H_6_ or silicon (111) substrates. In some instances both *cis*- and *trans*-Fe(SiCl_3_)_2_(CO)_4_ has been used as the single-source organometallic precursor (SSP) for iron and silicon (Figure 11). Some of the organometallic systems that produce FeSi have also been reported to produce FeSi_2_. Most of the examples occur by either chemical vapor deposition or vapor-phase or solid-phase epitaxy processes, thereby belonging to the VLS-type reactions.

The first example of organometallic chemical vapor deposition (OMVCD) of iron silicides appeared from Dormans in 1991 [77]. Dormans discovered that the decomposition of ferrocene alone in vacuum or in an inert gas occurred by auto-reduction of the metallocene yielding the initial reduction product cyclopentadienyl radical (C_5_H_5_**^.^**) which could recombine to dihydrofulvalene (C_10_H_10_) [78] or disproportionate heterogeneously, leading to the incorporation of carbon in the growing Fe layer obtained in the OMVCD process. However, when the decomposition of the ferrocene was performed in a H_2_ atmosphere, the initial reaction products were observed to be relatively stable volatile hydrocarbons, namely, cyclopentadiene, cyclopentene and cyclopentane. Thus, the growth of Fe layer from ferrocene in H_2_ did not lead to carbon incorporation. In order to form the iron silicide, FeSi, a silicon precursor such as Si_2_H_6_ was supplied to the reactor along with ferrocene in H_2_. Both Auger sputter depth profiling and Rayleigh backscattering studies revealed that the bulk of the grown layers contained approximately 10 at % Fe, 60 at % Si and 30 at % C nearly independent of the deposition conditions. In the presence of Si_2_H_6_, the cyclopentadienyl groups were found to be not inert and were found to incorporate some carbon. The independence of the film composition to the process conditions from kinetic growth studies suggested that the deposition took place via an intermediate. It was assumed that in this intermediate the Cp ring was no longer directly attached to the metal by the relatively weak π-δ interaction but was now bonded to the metal or silicon by a much stronger σ-bond, so that the organic residue was not released easily. The XRD spectra of the iron silicide layers on non-silicon substrates revealed that at 700 °C the deposit was amorphous. At 800 °C the mobility of the atoms was found to increase and polycrystalline FeSi was observed to form.

In an example using a SSP for FeSi formation, Zybill *et al.* employed Fe(SiCl_3_)_2_(CO)_4_ for the formation of polycrystalline cubic FeSi films at 350*–*500 °C by low pressure chemical vapor deposition. *cis*-Fe(SiCl_3_)_2_(CO)_4_ was synthesized by the reaction of dodecacarbonyltriiron with trichlorosilane in the absence of any solvent at elevated temperatures (120 °C, 36h) in a sealed Carius tube (Equation 1) [79]. The films were deposited in a specially constructed hot-wall reactor containing Pyrex-glass substrates. The FeSi/glass films were polycrystalline with crystallites of 100 to 300 nm average diameter. The crystallites were found to form globular aggregates of about 800 nm–l.2 μm diameter which were interconnected to form a porous 3-D network (porosity 0.41). The thickness of the investigated films varied from 2.0 to about 20.1 μm. However, when the deposition was obtained on (100)Si substrates, (001) oriented columnar films of orthorhombic *β*-FeSi_2_ were formed, which will be discussed in the following section [80]. Thus, the nature of the substrate was seen to dictate the type of the iron silicide formed by this VLS-type CVD process.


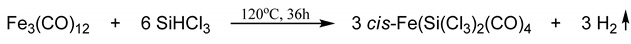
(1)

**Scheme 2:**
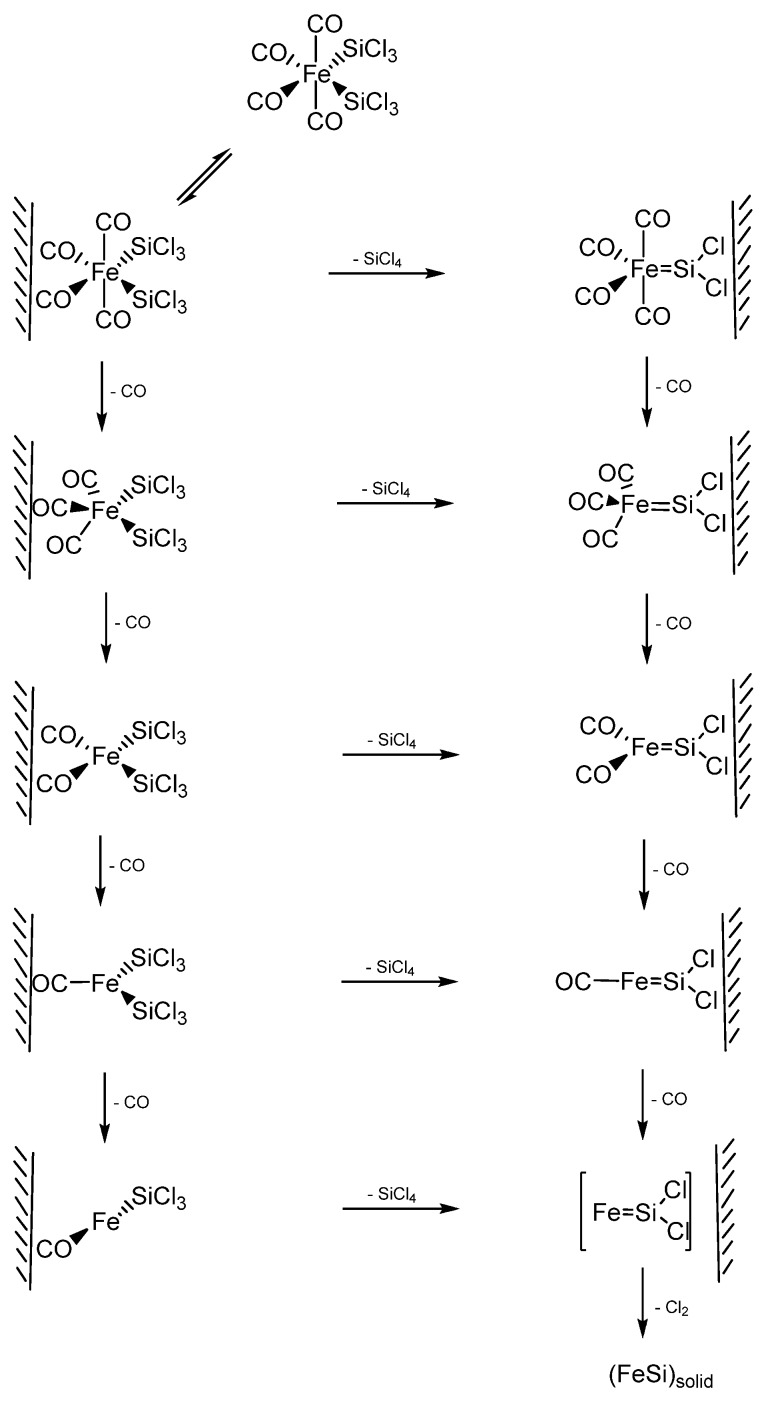
The two cascading reactions resulting from the initial CO or SiCl_4_ elimination from *cis*-Fe(SiCl_3_)_2_(CO)_4_ to produce FeSi films. Adapted from Ref. [83] with permission from American Chemical Society.

Zybill *et al.* further investigated the reaction mechanism of formation of FeSi films by the above CVD reaction of *cis*-Fe(SiCl_3_)_2_(CO)_4_ using *in situ* photoelectron (PE) spectroscopy in the surface-controlled regime up to 600 °C [80]. The experimental data provided evidence for SiCl_4_ elimination. The reaction was assumed to occur at the surface via adsorbed silylene complex intermediates. The spectra, at pyrolysis (surface) temperatures of 400*–*600 °C, were found to exhibit moderately intense ionizations from the CO *n*- and *π*-orbitals and intense ionizations from the SiCl_4_ 1t_1_, 3t_2_, 1e, 2t_2_, and 2a_1_ orbitals. In addition, a small peak at 18.08 eV was recorded resulting from the *n*-bonding electrons of CO_2_, which was formed from CO via the Boudouard equilibrium. No further products were observed in the entire temperature range; particularly not SiHCl_3_ [81], Cl_3_SiSiCl_3_, Cl_2_, Cl, COCl_2_, or COClH. The selective elimination of SiCl_4_ and CO was confirmed by an independent pyrolysis experiment in a mass spectrometer. In addition, a SiCl_4_ molecular peak with its fragmentation pattern was observed in the mass spectrometer at a temperature of 190 °C. These results suggested a decomposition pathway of *cis*-Fe(SiCl_3_)_2_(CO)_4_ via elimination of SiCl_4_ and formation of multiply bonded highly reactive intermediate silylene species such as [Fe(CO)_4_=SiCl_2_] [82]. It was pointed out that, under the chosen reaction conditions, the reaction most likely took place at the surface. In such a scenario, the authors opined that an intermediate such as [Fe(CO)_4_=SiCl_2_] will remain adsorbed and, after further elimination of CO and Cl_2_, undergoes nucleation to finally give polycrystalline FeSi [83]. A complete mechanistic scheme was further proposed presumably involving two cascades of either CO or SiCl_4_ elimination reactions (Scheme 2), both of which were in accordance with the experimental data. Furthermore, the Cl migration from Si to Fe (after loss of CO) appeared as a possibility even though it was energetically less favored. The authors reported that the direct observation of highly reactive surface species such as [Fe(CO)_4_=SiCl_2_]_surf_, [Fe(CO)_3_=SiCl_2_]_surf_, and [FeCl(CO)_3_=SiCl_2_]_surf_ was the subject of prevailing research efforts at the time of their report.

**Figure 12:**
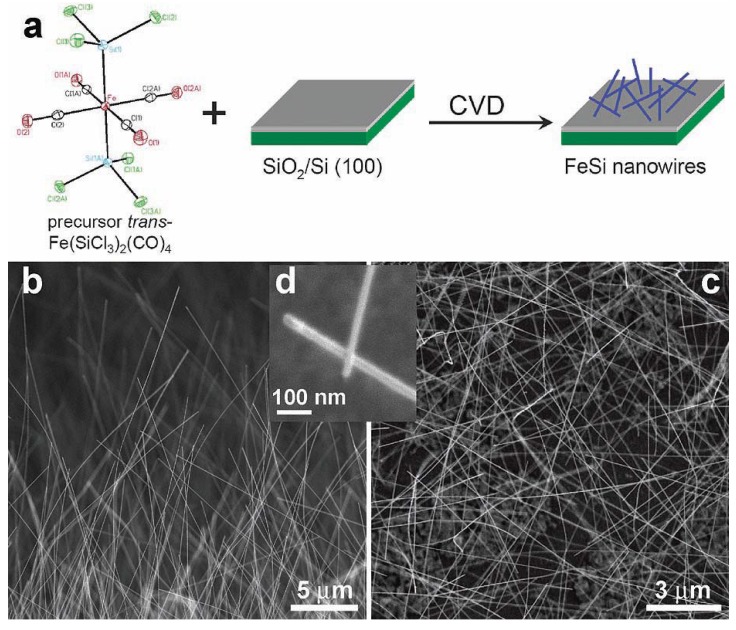
(a) FeSi NW growth from single-source precursor *trans*-Fe(CO)_4_(SiCl_3_)_2_. Representative SEM images of FeSi NWs: (b) over the edge of the growth substrate; (c) over the substrate; and (d) a close up view highlighting the NW tips. Reproduced from Ref. [84] with permission from American Chemical Society.

Jin *et al.* [84] reported the first examples of the chemical synthesis of free-standing single-crystal nanowires (NWs) of FeSi, which, even though debated, is the only known transition-metal Kondo insulator and has the host structure for ferromagnetic semiconductor Fe_x_Co_1-x_Si (Figure 12). The authors achieved this by the simple chemical vapor deposition of the SSP compound *trans*-Fe(SiCl_3_)_2_(CO)_4_. The SSP *trans*-Fe(SiCl_3_)_2_(CO)_4_ was synthesized from Fe_3_(CO)_12_ and SiHCl_3_ following a procedure modified from that of Zybill *et al.* in Equation 1 [79]. The product was spectroscopically identified and structurally characterized to be *trans*-Fe(SiCl_3_)_2_(CO)_4_ (Figure 12). The air- and water-sensitive yellow crystalline solid, *trans*-Fe(SiCl_3_)_2_(CO)_4_, could be handled briefly in air during nanowire synthesis setup and was found to readily sublime at moderately low temperatures. It was opined that compared with conventional multisource metal organic CVD, the SSP approach allowed simpler and safer experimental setups due to the elimination of highly hazardous liquid precursors (such as Fe(CO)_5_ typically for Fe and SiCl_4_ for Si), and further, allowed easier and precise control over stoichiometry, and higher quality material growth [85,86]. Thus, SSPs can be a solution to the typical problems confronted in the conventional multisource CVD processes. It is interesting to note here that these SSP-derived CVD reactions may be considered to be the vapor phase equivalent of the SLS-type reactions wherein the solid matrix is produced from a single source polymer precursor (*vide supra*).

**Figure 13:**
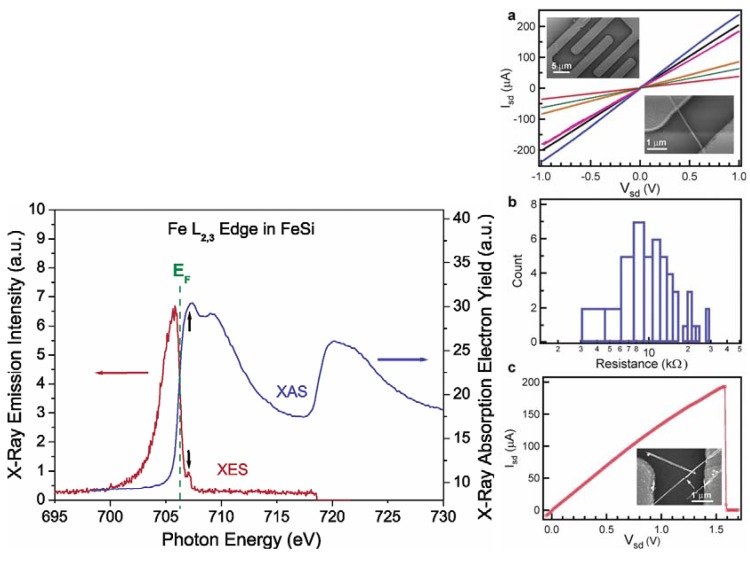
(Left) Spectroscopic characterization of FeSi nanowires by X-ray absorption spectroscopy (XAS) and X-ray emission spectroscopy (XES) at the Fe *L*_2,3_ edge. The black arrows point to the excitation energy (top) and the elastic peak (bottom). (Right) Room-temperature transport properties of FeSi NWs in two-terminal devices. (a) *I*_sd_ vs *V*_sd_ for several typical FeSi NW devices. (Insets) SEM images of a typical device. (b) Histogram of observed resistance for 47 single FeSi NW devices. (c) *I*_sd_ vs *V*_g_ recorded for a typical FeSi NW device that breaks down at higher voltage and current. (Inset) SEM image of this particular device after failure. The arrow highlights the breaking point. Reproduced from Ref. [84] with permission from American Chemical Society.

Jin *et al.* achieved the synthesis of the FeSi NWs in a home-built CVD setup comprised of a quartz tube heated by a tube furnace and equipped with pressure and gas flow controls. The SSP was sublimed at the upstream end of the tube, while Si/SiO_2_ substrates were placed in the hot zone of the heated tube. Straight and smooth FeSi nanowires were produced on silicon substrates covered with a thin layer of silicon oxide. This modified VLS process which did not use a separate metal catalyst produced FeSi NWs that had no catalyst tips. Such catalyst tips are typically observed in catalyst- initiated or catalyst-patterned VLS processes [84]. The structures of the nanowires were found to depend strongly on the surface employed. The authors discovered that the key to a successful CVD growth of FeSi NWs was to use silicon substrates covered with a thin layer of silicon oxide of thickness between 1 and 2 nm, which could be prepared by simply exposing a HF etched silicon substrate in ambient air at room temperature for 7*–*10 days, or in a “metal etch” solution (30% H_2_O_2_: 37% HCl: H_2_O; 1:1:5 v/v) at 70 °C for 10 min. Representative scanning electron microscopy (SEM) images (Figure 12b-d) revealed smooth and straight NWs of 10*–*80 nm in diameter and tens of micrometers in length produced in high density by this simple approach. More uniform diameter distribution (10 nm) was observed within each synthesis but a wider distribution was observed for NWs from different preparations. X-ray absorption and emission spectroscopies were used to verify the identity of FeSi NWs (Figure 13). Room-temperature electrical transport measurements using NW devices showed an average resistivity of 210 *μ*Ω cm, similar to the value for bulk FeSi (Figure 13 a-c). It was opined that this unique synthetic approach to FeSi NWs will be generally applicable to many other transition-metal silicides.

Three other reports of production of nanoscale FeSi involve either organometallic vapor-phase or solid-phase processes that fall in the class of VLS-type reactions, with the growth platform being a Si(111) surface. In the first one, Andre *et al.* reported the production of iron silicide thin films on silicon (111) substrates using the metalorganic vapour phase epitaxy (MOVPE) process with iron pentacarbonyl (Fe(CO)_5_) and disilane (Si_2_H_6_) precursors [87]. For the typical growth of *ε*-FeSi, the Si wafer was introduced in the reactor, just before the iron silicide growth, and *in situ* silicon deoxidation was achieved. The process consisted of annealing the wafer at 1040 °C for 5 min under a hydrogen flow. The substrate temperature was then stabilized at 540 °C to facilitate the deposition of *ε*-FeSi by injecting Fe(CO)_5_ and Si_2_H_6_ diluted in a large hydrogen flow at a Si/Fe ratio of 0.5. Continuous and dendritic zones of the product were observed in the lattice image and diffraction pattern taken along the Si(110) direction. The thin film of *ε*-FeSi had a thickness of 12 nm and contained 50 nm sized crystallites. It was also observed that *ε*-FeSi was unstable on a Si(111) substrate at high temperatures (800*–*850 °C) as it converted to *β-*FeSi_2_. The authors further probed the MOVPE process based on the reaction between Fe(CO)_5_ and Si_2_H_6_ in the temperature range 520*–*560 °C under a hydrogen atmosphere. In a codeposition mode, below 520 °C, no reaction was observed between Fe(CO)_5_ and Si_2_H_6_. However, above 560 °C, the formation of iron and silicon oxides was observed. Thus, the addition of disilane in the gas phase was found to increase the stability of Fe(CO)_5_ and prevent its decomposition upstream of the rotating graphite susceptor, the site of the epitaxial growth. This, incidentally, is similar to the observation of Dormans on the stability of the cyclopentadienyl groups in ferrocene in the presence of Si_2_H_6_ [77]. Based on these observations, the following mechanism was proposed for the growth of FeSi film. It was postulated that at 540 °C, on a clean Si surface, Fe(CO)_5_ and Si_2_H_6_ in a hydrogen gas flow initially produced a bcc-Fe deposition. The iron was then believed to diffuse into the silicon substrate to produce *ε*-FeSi. When a thin layer of *ε*-FeSi was obtained, homoepitaxial growth of *ε*-FeSi was believed to happen resulting in the final product film. It was suggested that the mechanism was in perfect agreement with the following observations. (a) Fe deposition on sapphire appeared at 540 °C with Fe(CO)_5_ and Si_2_H_6_ mixture in hydrogen gas flow and no *ε*-FeSi phase was detected by X-ray diffraction. (b) TEM observations indicated that the first *ε*-FeSi material was localized in the silicon substrate and then obtained by diffusion; afterwards the homoepitaxy was observed. (c) Finally, by solid-phase epitaxy Fe and Si were found to generate *ε*-FeSi in the same temperature range. Due to the absence of structural data on the Fe-Si precursor at 540 °C, the authors postulated that some Fe-Si bonded species were present at this temperature due to the displacement of CO ligands which was a driving force for the formation of *ε*-FeSi.

**Figure 14:**
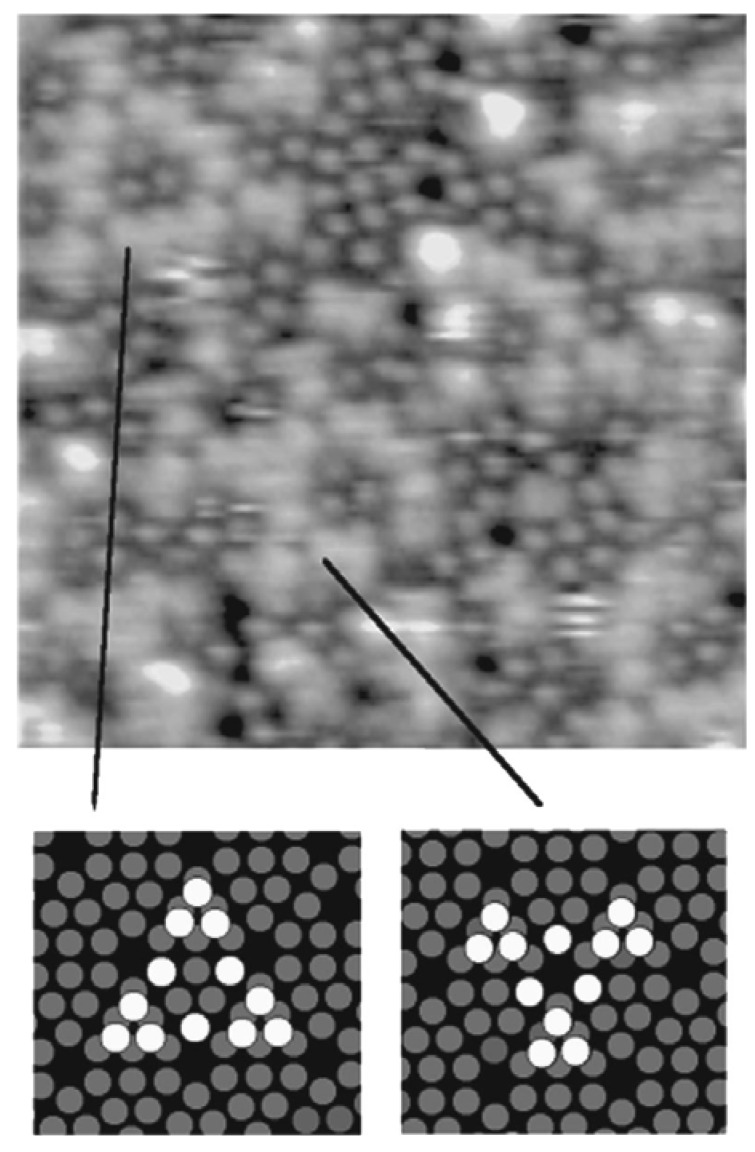
Characteristic structures formed by Fe(CO)_5_ adsorption on Si(111)7×7 (15 nm)^2^ as obtained from STM studies. Grey ring: Si(111)7×7 adatoms, White ring: adsorption sites. Reprinted with permission from Ref. [88]. Copyright 1998, American Vacuum Society.

In a study by Thibaudau *et al.*, a comparison of room temperature adsorption reactions of Fe(C_5_H_5_)_2_ and Fe(CO)_5_ on Si(111)7×7 and B/Si(111)√3×√3 R30° has been reported [88]. On Si(111)7×7, the adsorption sites were identified by means of scanning tunneling microscopy (STM). The authors proposed a Fe(C_5_H_5_)_2_ adsorption model on Si(111)7×7 consisting of a di-σ bridging by the molecule between an adatom and a restatom site similar to that proposed for the ethylene. This process was found to be in agreement with the lack of reactivity of this molecule on the B/Si(111)√3×√3 R30° surface. For the Fe(CO)_5_, the evidence was found of a dissociative adsorption on nucleophilic sites. Thus, at higher temperatures, Fe(CO)_5_ exposure to the Si(111)7×7 and B/Si(111)√3×√3 R30° surfaces was found to produce good quality iron silicide. However, a similar exposure of ferrocene was found to produce only silicon carbide. The preferential formation of the films, particularly on the Si(111)7×7 surface, was explained in terms of the chemisorption process that occurred in each case at room temperature. It was argued that as the iron atom in each molecule is located within cavities created by surrounding ligands, Cp or CO, the adsorption can only occur through interaction of the substrate with the ligands. In the case of Fe(C_5_H_5_)_2_, two Si–C covalent bonds were proposed to have been created. The binding energy of the substrate (Si(111)7×7 surface) with the ligand (~2 × 3.9 eV) [89] was known to be greater than the cohesion energy of Fe(C_5_H_5_)_2_ (6.16 eV) [90]. This facilitated the dissociation of Fe(C_5_H_5_)_2_ at the surface at high temperatures. The decomposition of the Cp ring would then lead to the formation of carbide. However, in the case of Fe(CO)_5_, the binding energy between the Si(111)7×7 surface and the ligand (CO) was found to be weak, and as a result, at high temperatures, Fe(CO)_5_ will not adsorb strongly on the surface. Since only a stronger interaction between the surface and Fe(CO)_5_ would have allowed the adsorption of Fe(CO)_5_, the only possible way to achieve such an interaction would necessitate the access of the Fe atom by a dissociation of the Fe(CO)_5_ molecule. This process started only above 140 K, above which Fe(CO)_5_ adsorbed onto the surface by the sequential dissociation of CO ligands to finally form FeSi according to the Equation 2. Thus, the authors reasoned that in contrast to Fe(C_5_H_5_)_2_, the adsorption of Fe(CO)_5_ does not happen through the ligand, but rather through the Fe atoms at the surface after a complete dissociation of the CO ligands from the molecule. The characteristic adsoption sites were also reported of Fe(CO)_5_ on the Si(111)7×7 surface as obtained from STM studies (Figure 14). This resulted in the growth of a good quality FeSi film instead of the SiC film as in the case of Fe(C_5_H_5_)_2_.


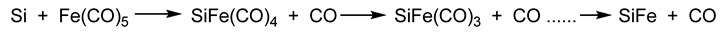
(2)

Zanoni *et al.* reported a similar study of the exposure of a Si(111)7×7 surface with Fe(CO)_5_ in the presence of unmonochromatized synchrotron radiation ("white beam"), which leads to the formation of FeSi [91]. The process was followed by photoemission spectroscopy of the core and valence levels of Fe(CO)_5_ and the surface. A FeSi layer was obtained, as a result of partial decomposition of Fe(CO)_5_. The quality of the silicide layer obtained in this study in comparison to that obtained by solid phase epitaxy in the Thibaudau *et al.* example was found to be very poor, and was attributed mostly to the incomplete dissociation of CO ligands of Fe(CO)_5_ under the utilized white beam flux and the exposure time. It was estimated that under conditions of their experiment, only 50% of the Fe(CO)_5_ was completely dissociated by the white beam. The molecular states of the unexposed Fe(CO)_x_ were found to have band states with a clear Fermi edge in the valence band photoemission spectra. On the other hand, the Fe that was fully dissociated from Fe(CO)_5_ was found to interact with the silicon substrate and display a silicide-like spectra. It was further determined that the kinetics of the silicide nucleation was heavily influenced by the large amount of chemical species at the interface, due to the partial decomposition of the Fe(CO)_5_ molecules. Silicon carbide and oxide spectra were also observed in the product.

### 2.5. Organometallic reactions that produce nanomaterials of FeSi_2_

Among the Fe silicide series, the majority of examples of a Fe silicide nanomaterial that is derived from an organometallic compound are that of *β*-FeSi_2_, even though thermodynamically *ε*-FeSi is more stable than *α*− or *β*−FeSi_2_. Interestingly, in solid phase reactions involving Fe thin films and Si substrates, the iron silicide phase formation has been observed to depend not only on the time and temperature of the heat treatment but also on the thickness of the initial layer and on the formation mode. As a function of the thickness, typically two phases appear: *ε*-FeSi and *β*-FeSi_2_. The second phase, *β*-FeSi_2_ forms from *ε*-FeSi by a nucleation controlled reaction. Below a critical thickness of the product layer, the *β*-FeSi_2_ nuclei were found not to reach the critical radius, thus, thwarting a continuous growth of *β*-FeSi_2_. As a result, *ε*-FeSi phase is found in thinner samples and *β*-FeSi_2_ phase in thicker films [92]. Furthermore, the thermodynamically stable *ε*-FeSi has been shown to be unstable on a Si(111) substrate at high temperatures (800–850 °C) and has been found to convert slowly into the *β*-FeSi_2_ phase. Similarly, the *α*-FeSi_2_ phase has also been found to convert to the *β*-FeSi_2_ phase at high temperatures. These observations from solid-state reactions are worth considering when studying the examples of *ε*-FeSi, *α*-FeSi_2_ and *β*-FeSi_2_ formed from various epitaxy processes involving organometallic compounds on Si(111) substrate discussed in this section and in the previous section [88]. Additionally, as in the report by Andre *et al.*, all *ε*-FeSi, *α*-FeSi_2_ and *β*-FeSi_2_ have been shown to grow at 540 *°*C through the control of the Fe to Si ratio, by varying the reacting equivalents of Fe(CO)_5_ and Si_2_H_6_ during MOVPE process [87]. Furthermore, all of the reported productions of nanoversions of *α*− or *β*-FeSi_2_ discussed in this section fall under the VLS-type reactions.

The first example of the production of FeSi_2_ from an organometallic species appeared in 1977 from Aylett and Colquhoun [93]. The pyrolysis of [Fe(CO)_4_(SiH)_3_]_2_ at 773 K in a flow system was found to afford *β*-FeSi_2_. Flow pyrolysis of [Fe(CO)_4_(SiH)_3_]_2_ at 773 K with helium as carrier gas gave rise to a dark grey film on the surface of a silica substrate, and a ring of brittle material around the tip of the inlet nozzle. Both types of deposit were crystalline; the nozzle deposits in particular were found to produce sharp X-ray powder-diffraction patterns which confirmed the presence of *β*-FeSi_2_. X-Ray patterns from the substrate deposits were more diffused, but showed that they contained the same phases as the corresponding nozzle deposits. The morphologies of the nozzle and substrate deposits were examined by scanning electron microscopy which revealed extensive dendritic growth in the former but a uniform blastular texture in the latter, characteristic of many materials formed by rapid vapor deposition. The diameter of the dendritic *β*-FeSi_2_ was found to vary from 100 nm to several μm. It was suggested that the most important fragmentation pattern for silyl metal carbonyls involved the stripping of hydrogen and CO, leaving abundant [M_x_Si_y_]^+^, and Si^+^ ions for the production of the silicides [94,95]. Other than this example where FeSi_2_ was grown on a silica substrate, all other examples of FeSi_2_ growth involved CVD processes involving epitaxial growths on either Si(111), Si(110) or Si(100) substrate. The epitaxy processes included solid, vapor and gas phases.

**Figure 15:**
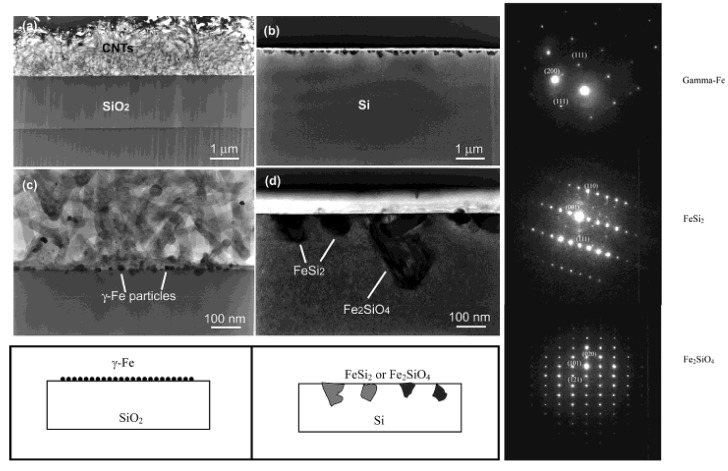
TEM images of the cross section of the substrates. (Left) (a) SiO_2_ area after CVD growth and removing of carbon nanotubes. (b) Si area without any nanotube growth but precipitate of submicron-size particles near the surface. (c) Enlarged picture from the nanotube/SiO_2_ interface in (a) showing the formation of gamma iron particles on silicon oxide surface and the growth of nanotubes from the particles formed. (d) Enlarged area from (b) showing the formation of iron silicide and iron silicate crystals during CVD processing. Corresponding schematics are also illustrated. (Right) The panels on the right hand side depicts, selective area diffraction patterns from the particles on the oxide surface (c) and on the Si surface (d), indicating gamma iron (fcc-Fe) formation and iron silicide and iron silicate formation during the CVD process on silicon oxide surface and silicon surface, respectively. Reproduced from Ref. [96] with permission from American Chemical Society.

Before discussing such examples, a unique example needs to be mentioned relating to the formation of multiwalled carbon nanotubes (MWNTs) and its connection to FeSi_2_. Ajayan *et al.* reported a strong selectivity for growth of MWNTs during the chemical vapor deposition using the vapor phase delivery of Fe(C_5_H_5_)_2_ catalyst precursor on patterned SiO_2_/Si substrates [96]. It was found that active Fe catalyst (*γ*-Fe or fcc-Fe) particles were formed on the SiO_2_ portion of the surface leading to the formation of highly aligned nanotubes on this portion of the substrate. However, in the Si regions, stable FeSi_2_ and Fe_2_SiO_4_ particles (both with particle sizes of 50*–*100 nm) were observed to have formed due to chemical reactions between the silicon surface and the Fe particles at high temperature (Figure 15). This was found to lead to an inhibition of nanotube growth in the Si regions [96]. Even though the impetus for this work was not the formation of an iron silicide, it demonstrated that FeSi_2_ was not a good catalyst for the growth of carbon nanotubes. This is in line with the previous report that FeSi is not as good a catalyst as Fe for carbon nanotube formation [97]. In light of this, it is suggestive that the formation of carbon nanocapsule around Fe_5_Si_3_ nanoparticles as in the work of Kolel-Veetil *et al.* [8] is due to the enhanced Fe concentration in Fe_5_Si_3_ compared to that in FeSi_2_. Thus, it can be expected that as the Fe content increases in a Fe silicide, the catalytic activity of the silicide for carbon nanotube formation should also increase. Hence, the catalytic activity for carbon nanotube formation should diminish along the following series: Fe_3_Si > Fe_5_Si_3_ > FeSi > FeSi_2._

As mentioned in the previous section, the report of Andre *et al.* on the production of iron silicide thin films on silicon (111) substrates using the MOVPE process with iron pentacarbonyl (Fe(CO)_5_) and disilane (Si_2_H_6_) precursors also included the production of *α*-FeSi_2_ and *β*-FeSi_2_ thin films [87]. The deposition of *α*-FeSi_2_ or *β*-FeSi_2_ thin film occurred by the injection of Fe(CO)_5_ and Si_2_H_6_ diluted in a large hydrogen flow at a Si/Fe ratio of 1 to 4 onto a substrate that was stabilized at 540 *°*C, without any modification of the layer properties. Continuous and dendritic zones of the product were observed in the lattice image and diffraction pattern taken along the Si(110) direction. The thin film of *α*-FeSi_2_ had a thickness of 5 nm and contained 5 nm high and 20 nm large-sized crystallites. In comparison, to grow thin films of *β*-FeSi_2_ with a thickness of 100 nm and containing 10 nm high and 100 nm large-sized crystallites, annealing at 720–800 °C of ε−FeSi and *α*-FeSi_2_ layers grown on Si(111) was found to be required. The annealing process lead to the formation of polycrystalline *β*-FeSi_2_. The thermoelectric and direct band gap characteristics of the thick *β*-FeSi_2_ were also investigated. The Seebeck effect, defined as the potential variation generated by a temperature gradient between two points in a material, was performed on thick *β*-FeSi_2_ wafers in the range between 30–110 *°*C. The relationship was found to be linear and the coefficient was found to be as high as 370 μV/K. The photoluminescence spectrum at 4K of the *β*-FeSi_2_ wafers was found to exhibit a single peak with energy in the range between 0.8 and 0.85 eV.

Schafer *et al.* reported the epitaxial growth of iron disilicide thin layers on Si(111) surface by gas source molecular beam epitaxy (GSMBE) in the temperature range 450–550 *°*C [98]. Fe(CO)_5_ and SiH_4_ were used as beam sources and separately injected into the growth chamber through glass capillary arrays focused onto the heated Si(111) surface. The reactant gases were found to decompose on the surface at temperatures between 450 and 550 *°*C. The Si surface was heated by means of direct current flow through the sample. No traces of carbon incorporation in the as grown layers were revealed within the sensitivity of the employed *in situ* techniques. The growth phases were characterized *in situ* by means of high-resolution electron energy loss, ultraviolet and x-ray photoelectron spectroscopies. The formation of an epitaxial metallic *γ*-FeSi_2_ layer at the interface with the silicon substrate was revealed and no complete relaxation of this strained metastable interface layer was observed as the growth proceeded to yield the semiconducting equilibrium *β*-FeSi_2_ phase. The authors confirmed the coexistence of the heterostructures of the metallic (CaF_2_) and semiconducting (orthorhombic) FeSi_2_ structures in the GSMBE-grown structures by cross-section transmission electron microscopy.

The report by Zanoni *et al.* of the production of FeSi layer by the exposure of a Si(111)7×7 surface with Fe(CO)_5_ in the presence of unmonochromatized synchrotron radiation, discussed in the previous section, also contained the study of the valence band spectra of a bcc-Fe film, of the metalloorganic deposit from Fe(CO)_5_ after exposure to the white beam, and of the Fe/Si(111)7×7 interfaces, prepared by the metalloorganic (MO) method using synchrotron radiation and solid-phase epitaxy (SPE) (chemical vapor deposition) method after annealing at 300 °C and at 600 °C [91]. At the SPE interface, the metallic *α*-FeSi_2_ nucleated at 300 °C was found to convert to the semiconducting *β*-FeSi_2_ at 600 °C as evident from photoemission spectroscopy of core and valence levels of the silicides. The density of states of the MO surface, on the contrary, was found to become increasingly metallic after annealing at 300 °C and to exhibit new states between 1.5 and 2.5 eV of binding energy at 600 °C, along with an intense oxide peak at 6 eV. Thus, it appeared that the MO interface was more heterogeneous than the SPE interface and was affected by contamination. This was attributed to the incomplete decomposition of Fe(CO)_5_ by the white beam of the synchrotron radiation.

Chen *et al.* reported the solid phase epitaxy (SPE) growth of epitaxial *β*-FeSi_2_ films by using Fe/Si(100) heterostructures obtained by metalorganic chemical vapor deposition (MOCVD) of Fe(CO)_5_ [99]. The formation of the *β*-FeSi_2_ thin films included the initial deposition of Fe on Si(100) by MOCVD and a subsequent annealing. Before deposition, the silicon substrates were baked in high purity H_2_ for 20 min at high temperatures to provide a clean surface. High purity H_2_ was also used as the carrier gas. The deposition temperature was 170 °C and the Fe(CO)_5_ source was kept at 5 °C. The sample was annealed in H_2_ ambient at 170 °C for 10 min after deposition. The deposition rate was 14 Å/min. The highly textured Fe layer deposited on the silicon substrate was tested by x-ray diffraction (XRD) and electron microscopy techniques. Such Fe/Si samples were annealed in a N_2_ ambient at 600 °C for 2 h. The *β*-FeSi_2_ film was found to successfully form after the annealing process. Optical transmission measurements studies by the authors revealed a strong absorption of *β*-FeSi_2_ near 0.87 eV.

In the example reported by Zybill *et al.* mentioned in the previous section, the low pressure chemical vapour deposition of the single source precursor Fe(SiCl_3_)_2_(CO)_4_ at 350*–*500 °C on (100)Si substrates, resulted in the formation of (001) oriented columnar films of orthorhombic *β*-FeSi_2_ [80]. It was observed that *β*-FeSi_2_ film grew on (100)Si with the (010) or (001) direction parallel to the (011) direction of Si with only 1.5 or 2.1% misfit, which allowed minimization of interfacial stress and strain. The films were characterized by X-ray photoelectron spectroscopy, X-ray diffraction, scanning electron microscopy and atomic force microscopy [80]. The films of FeSi_2_/(100)Si showed a very uniform surface with a grain size of about 200 nm. The average thickness of the films, grown as a result of surface controlled process, was about 3 μm. XRD measurements proved the crystallites to be uniquely (100) oriented. The authors reasoned that these results clearly demonstrated the dominant influence of the substrate on the film growth. In the case of an irregular glass surface, a randomly oriented crystalline material of high porosity was obtained. For the (100)Si substrate, an imprint of the (100)Si surface onto the FeSi_2_ film was achieved, which induced the formation of a completely (100) oriented epitaxial FeSi_2_ film. AFM studies of the FeSi_2_/Si films were found to reveal the uniformity of the films, which showed grains with lateral dimensions of about 200 nm. The maximum height difference was measured to be 112 nm. For the FeSi/glass films, a granular structure of about 1*–*3 μm size was observed with a maximum height difference of 2.19 pm. The AFM needle did not penetrate deeper into the channels of the porous material. A four-point probe resistivity measurement was found to yield a room temperature resistivity of 6.0 × 10^4^ μΩ cm for FeSi_2_/Si, which is in the typical range for a semiconductor.

In a series of recent papers [100,101,102], Akiyama *et al.* have described the synthesis of *β*-FeSi_2_ by epitaxial growth on Si(111) or Si(100) substrate by MOCVD. They were interested in forming stoichiometric *β*-FeSi_2_ films, in light of the report by Takakura *et al.* of the existence of a wide range of nonstoichiometry in *β*-FeSi_2_ (in the form of *β*-FeSi_2-x_) thin films that were formed on Si(100) by large ion-pumped molecular beam epitaxy system [103]. The nanometer-thick iron-silicon multi-layers were deposited on a *β*-FeSi_2_ template, which was epitaxially grown on a high-resistance Si(100) substrate, with subsequent annealing. Moreover, a change in the conduction type, i.e, p- or n-type, of the films was reported based on the Si/Fe ratio in the films. This was surprising considering the fact that *β*-FeSi_2_ exists almost as a line compound with no wide range of non-stoichiometry in the Fe-Si phase diagram [12,13,14]. Akiyama *et al.* utilized SiH_4_ and Fe(CO)_5_ as the Si and Fe sources, respectively, in the MOCVD process. Fe(CO)_5_ source sealed in the vessel was mixed with H_2_ gas and was carried to the reactor simultaneously with SiH_4_. The composition of the films formed at 700*–*800 °C revealed that the constituent phase was indeed the stoichiometric *β*-FeSi_2_ in good agreement with the Fe–Si phase diagram. Any local excess of Fe in the growing *β*-FeSi_2_ film was seen to be neutralized by the diffusion of Si into the film from the substrate. A cessation of the diffusion was observed when *β*-FeSi_2_ was formed as a single phase, as evident from the stoichiometry of *β*-FeSi_2_ [100,101]. The grains in the film were found to be about 50 nm in size.

Akiyama *et al.* further explored the formation of *β*-FeSi_2_ films by their deposition at 750 °C using a simultaneous supply of Fe(CO)_5_ and SiH_4_ by MOCVD process [102]. It was observed that Fe(CO)_5_ decomposed in the gas phase prior to its arrival at the substrate surface. It was suggested that an intermediate reactant which was more stable than Fe(CO)_5_ was created from both Fe(CO)_5_ and SiH_4_ which facilitated the Fe–Si film formation. The epitaxial *β*-FeSi_2_ films that were obtained on Si(111) substrates were found to contain neither carbide nor oxide phases by XRD studies. The authors also studied the deposition rates of iron and silicon in relation to ratio of the reactants wherein the SiH_4_ gas flow rate was varied from 0 to 5 cm^3^/min under a constant supply of Fe(CO)_5_. The study revealed three clear regions of reactivity. It was observed that, below a flow rate of 0.25 cm^3^/min, hardly any iron deposition occurred, even though it has been proposed that Fe(CO)_5_ decomposed in the gas phase in this region [88]. In the region between 0.25 and 0.5 cm^3^/min, the iron deposition was observed to begin and the deposition rate was found to increase drastically as the SiH_4_ gas flow rate was increased coinciding with an increase from 1 to 3.5 of the Si/Fe atomic ratio. In the region above a flow rate of 0.5 cm^3^/min, the iron deposition rate was found to increase linearly with an increase in the SiH_4_ gas flow rate. These data were suggested to indicate the following reaction during the mixing of gaseous Fe(CO)_5_ and SiH_4_ (Equation 3). This reaction scenario was postulated based on the reports of the reactions of Fe(CO)_5_ with phosphine (PH_3_) or arsine (AsH_3_) by Cotton *et al.* [104] and Lewis *et al.* [105]. These hydrides were found to replace CO radicals during heating or UV illumination. The Fe(CO)_5-n_(SiH_3_)_n_ intermediate reactant was considered to be more thermally stable than Fe(CO)_5_, as it proceeded to deposit iron at a temperature of 750 °C.


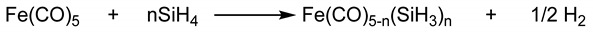
(3)

**Figure 16:**
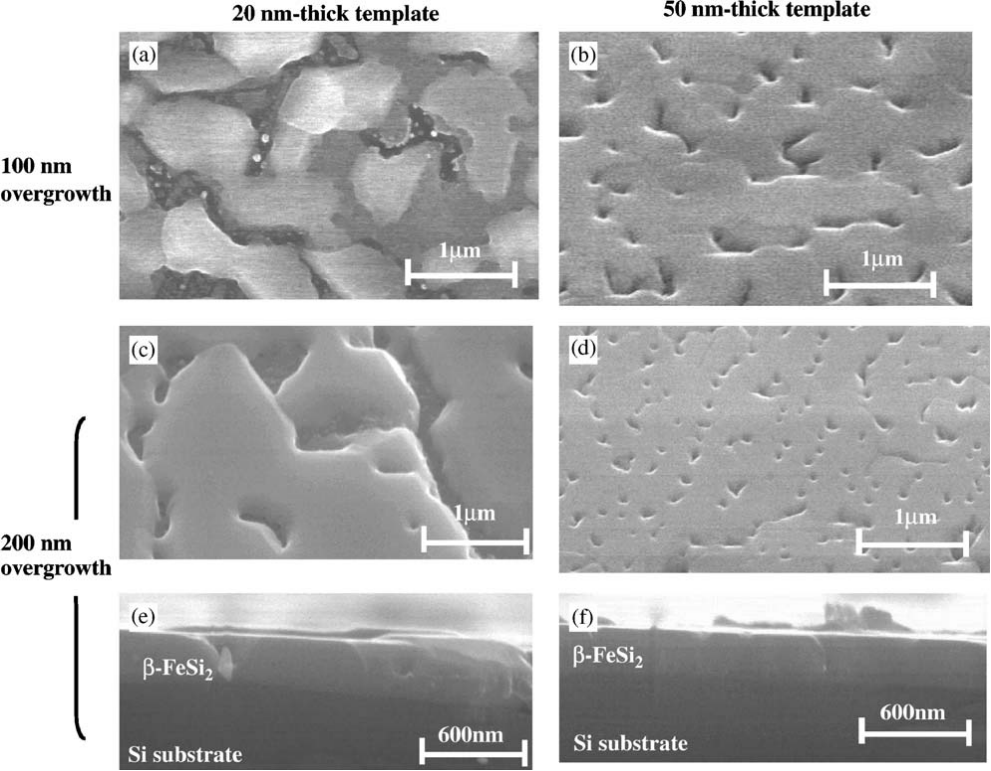
(a)–(d) Pane-view and (e) and (f) cross-sectional SEM images of MOCVD-overgrown films deposited on Si (111) substrates with ((a), (c), (e)) 20- or ((b), (d), (f)) 50-nm-thick templates; (a) and (b) after 100 nm MOCVD-overgrowth at 750 °C; (c)–(f): after 200 nm MOCVD-overgrowth at 750 °C. Reproduced from Ref. [106] with permission from Elsevier.

In two recent papers, Akiyama *et al.* studied the influence that a template layer had on the nature of epitaxially grown *β*-FeSi_2_ films by MOCVD. In the first paper, epitaxial (101)- and/or (110)-*β*-FeSi_2_ films with a continuous flat surface were grown on Si(111) substrates by MOCVD using a *β*-FeSi_2_ template layer with a high initial nuclear density [106]. With such a template layer, homogeneous grain growth was achieved resulting in the growth of flat surface films. For *β*-FeSi_2_ deposition on the template by MOCVD-overgrowth, Fe(CO)_5_ and SiH_4_ were used as iron and silicon sources, respectively. The Si/Fe atomic ratios of the films were maintained close to 2 by adjusting the input gas flow rates of the iron and silicon sources. Films grown on 50-nm-thick templates were observed to maintain a strong (101)- and/or (110)-orientation after MOCVD overgrowth and were found to have a full-width at half maximum (FWHM) of 0.591 for the rocking curve (Figure 16). The average roughness was 16 nm for a 200-nm-thick film grown on a 50-nm-thick template.

**Scheme 3:**
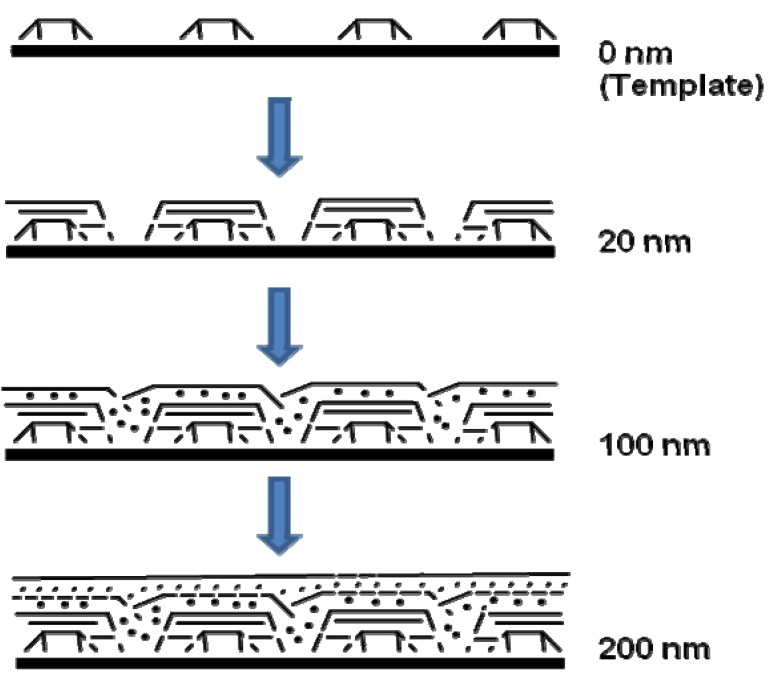
Schematic diagram of crystal growth of MOCVD-overgrown *β*-FeSi_2_ films on aggregated templates. Adapted from Ref. [107] with permission from Elsevier.

In the second paper, Akiyama *et al.* studied the characteristics of epitaxial *β*-FeSi_2_ thin films grown by MOCVD on Si(100) substrate having an aggregated *β*-FeSi_2_ template [107]. The horizontal growth in the templated *β*-FeSi_2_ films were found to produce smaller voids among islands during the overgrowth process thereby producing continuous films. The horizontal growth was attributed to the higher growth rate on the sidewall planes of *β*-FeSi_2_ than on its (100) plane. As in the first paper, Fe(CO)_5_ and SiH_4_ were used as iron and silicon sources, respectively, for *β*-FeSi_2_ deposition on the template by MOCVD-overgrowth. The authors suggested a mechanism for the MOCVD overgrowth on aggregated (100) *β*-FeSi_2_ template (Scheme 3). It was proposed that, during the early stage of MOCVD-overgrowth, the facet planes are present at the edges of aggregated *β*-FeSi_2_ grains. From the angle observed by the cross-sectional SEM image, these facet planes were assigned as (101) and (110) *β*-FeSi_2_ planes, with the angles to the (100) *β*-FeSi_2_ plane found as 51.81 and 52.01 degrees, respectively. Faster growth on these facet planes than on the (100) *β*-FeSi_2_ facets was considered to reduce the voids between islands, eventually leading to a continuous film. After a continuous film with smooth surface resulted from the coalescence of the grains, the MOCVD-overgrown film was considered to grow only vertically. In all papers by Akiyama *et al.*, no study was available on the electronic properties of the *β*-FeSi_2_ material.

**Figure 17:**
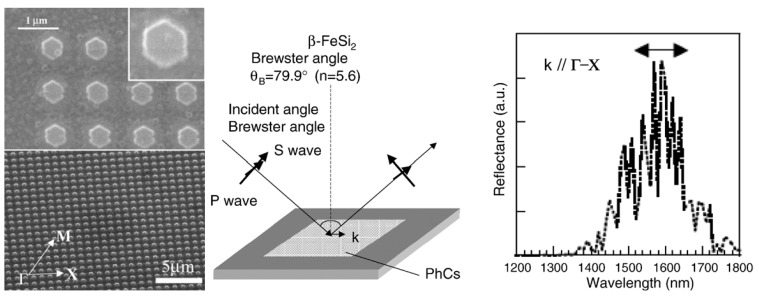
(Left) Square lattice photonic crystal with hexagonal columns of *β*-FeSi_2_ fabricated by an NLD-plasma etching process. (Right) Reflectance spectrum of polarized incident light at the Brewster angle. (Middle) A polarized light at the wavelength corresponding to the photonic band-gaps cannot propagate into the photonic crystal along any direction. Therefore, the reflectance becomes larger than the usual reflectance of the s-polarized light at the Brewster angle. Reproduced from Ref. [108] with permission from Elsevier Science.

Recently in two papers, Maeda *et al.* investigated the dry-etching properties of polycrystalline *β*-FeSi_2_ films that were formed by epitaxial growth on Si(111) by MOCVD using Fe(CO)_5_ and SiH_4_, in order to realize fabrication of photonic crystals (PhCs) that are several hundred nanometers in dimension [108,109]. It was observed that a higher reactive etching rate was obtainable using reactive ion etching with magnetic neutral loop discharge plasma (NLD), than by inductively coupled plasma (ICP). A two-dimensional PhC of *β*-FeSi_2_ was fabricated on Si substrates and its reflectance spectrum of incident polarized light was obtained to predict its photonic properties (Figure 17). The reflectance maximum in the spectrum was further analyzed to obtain the photonic band structures of the two-dimensional crystal with a square lattice of hexagonal *β*-FeSi_2_ columns (*n* = 5.6). Three photonic band-gaps for the transverse electric (TE) polarized light were obtained at frequency bands (a/λ) of 0.22–0.28, 0.38–0.48 and 0.59–0.66. At *a* (a lattice constant of *β*-FeSi_2_ column) = 1.0 μm, the photonic bands were observed in the three wavelength ranges of 3.57–4.55 μm, 2.08–2.63 μm and 1.52–1.69 μm. Therefore, it was established that the on-plane propagating vector k component of the p-polarized light in the wavelength range of 1.52–1.69 μm in air cannot exist in the crystal because any TE-polarized light propagation will be forbidden. Therefore, at the Brewster angle the reflectance was found to include s- and p-polarized light. The authors opined that this result showed that the two-dimensional *β*-FeSi_2_ square lattice can function as PhCs.

**Figure 18:**
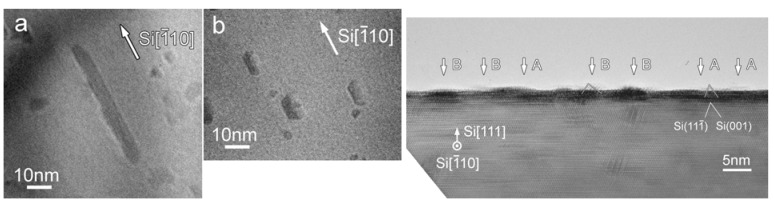
(Left) TEM micrographs of iron silicide nano-rods grown on a Si(111) substrate. (a) A nano-rod of high aspect ratio, and (b) nano-hexagons. Both types of rods grow parallel to the Si(110) direction. (Right) A TEM micrograph of iron silicide islands grown on a Si(111) edge plane at about 700 K. While islands A retain the plane spacings and plane angles of the Si substrates, islands B seems to have a different structure. Reproduced from Ref. [110] with permission from Springer Netherlands.

Finally, Tanaka *et al.* have reported the electron-beam induced chemical vapor deposition (EBI-CVD) of Fe(CO)_5_ on both Si(111) and Si(110) substrates at 673–873 K inside an ultrahigh vacuum transmission electron microscope [110]. The formation of iron silicide islands on both substrates was reported (Figure 18). Cubic silicide nano-rods were formed on Si(111) substrates by EBI-CVD with focused electron beams. The formation of *β*-FeSi_2_ islands was mainly observed on Si(110) substrates by EBI-CVD when the electron beam was broadly spread. It was shown that the size and the intensity of the electron beam played a significant role in EBI-CVD and affected the CVD process extensively [110].

The authors compared the mechanism of iron silicide growth using Fe(CO)_5_ during both conventional CVD and EBI-CVD processes. It was noted that, during the early stages of conventional CVD performed at elevated temperatures, Fe(CO)_5_ precursor molecules almost simultaneously reacted on dissociative Si adsorption sites (strong nucleophilic sites), resulting in the initial deposition of Fe clusters and subsequent formation of iron silicide islands [88]. However, at relatively low temperatures, Fe clusters formed by CVD tend to grow bigger, than clusters formed by typical Fe evaporation (a random deposition process), due to the autocatalytic effect in which previously deposited Fe atoms activate decomposition of both Fe(CO)_5_ and partially coordinated Fe(CO)*_x_* molecules incident on their surface through ligand exchange [111,112]. However, the authors reasoned that this may not play an important role at the high temperatures used in their study. It has been reported that epitactic growth of FeSi_2_ takes place in the phase sequence of cubic, α, and β with increasing Fe concentration [113]. Itakura *et al.* had reported that the lower temperature and the higher Fe concentration suppress the formation of *α*-FeSi_2_ and promote the formation of *β*-FeSi_2_ [114]. Thus, in the case of conventional CVD, fewer Fe atoms concentrate on the surface and formation of cubic iron silicide is observed. On the other hand, broad beam EBI-CVD drastically promoted the dissociation of Fe(CO)_5_ molecules [115]. It was reported that this dissociation does not only happen at the dissociation sites but also in the area where electron beams are irradiated above and on the surface. This caused the dissociated Fe atoms to diffuse to the nucleation sites (presumably to the dissociative adsorption sites) to contribute to the further growth of the silicide islands. All of this was suggested to imply a higher concentration of Fe atoms on the surface than during conventional CVD cases and hence, to the formation of *β*-FeSi_2_ islands, as frequently observed in their profile-view study.

## 3. Conclusions

It seems self evident from the foregoing sections that the research area involving the utilization of organometallics for the production of Fe silicides is still a nascent one; albeit one with an enormous potential for growth. In the synthesis of the room temperature ferromagnets, especially, there exist opportunities for the production of novel Fe and Si-containing polymers from which the iron-rich Fe_3_Si and Fe_5_Si_3_ nano-ferromagnets may be formed. In this regard, it is striking that the two examples each of formation of Fe_3_Si and Fe_5_Si_3_ nanomaterials from organometallic systems come from polymeric precursors of Fe and Si that are amenable to the formation of crosslinked networks and to their further pyrolysis for the product formation via SLS-type reactions. The lack of prevalence of VLS- and LLS-type reactions in the production of these two silicides makes one ponder whether these thermodynamically less-favored products including the metastable Fe_5_Si_3_ are not accessible by these types of reactions at all! It is, therefore, desirable to test this hypothesis by producing new polymeric systems of Fe and Si that are amenable to crosslinked network formation from which the formation of such iron silicides as FeSi and FeSi_2_ by SLS-type reactions may be coaxed. Even if such attempts were to fail, the selective spatial confinement of Fe and Si components in such developed systems should at least provide a controllable means to preferentially produce either Fe_3_Si or Fe_5_Si_3_ nanomaterials in large quantities. Furthermore, the underpinning effects that cause selectivity, based on the nature of the utilized Si(111), Si(110), Si(100), Si/SiO_2_ or pyrex glass substrate, among the thermodynamically-favored FeSi, *α*-FeSi_2_ and *β*-FeSi_2_ products produced in VLS-type reactions, such as in the epitaxial processes, should be further explored to delineate definitive trends. This should aid in the discriminitative formation of such iron silicides upon substrate selection. Finally, a broader development of comprehensive synthetic strategies for the production of new monomeric and polymeric organometallics containing Fe and Si that are targeted for the production of iron silicides is highly desirable for the advancement of this important area of electronic compounds.
